# Repetitive subconcussion results in disrupted neural activity independent of concussion history

**DOI:** 10.1093/braincomms/fcae348

**Published:** 2024-10-08

**Authors:** Kevin Grant Solar, Matthew Ventresca, Rouzbeh Zamyadi, Jing Zhang, Rakesh Jetly, Oshin Vartanian, Shawn G Rhind, Benjamin T Dunkley

**Affiliations:** Neurosciences and Mental Health, Hospital for Sick Children Research Institute, Toronto, ON, Canada M5G 0A4; Neurosciences and Mental Health, Hospital for Sick Children Research Institute, Toronto, ON, Canada M5G 0A4; Neurosciences and Mental Health, Hospital for Sick Children Research Institute, Toronto, ON, Canada M5G 0A4; Defence Research and Development Canada, Toronto Research Centre, Toronto, ON, Canada M3K 2C9; Faculty of Medicine, University of Ottawa, Ottawa, ON, Canada K1A 0K6; Defence Research and Development Canada, Toronto Research Centre, Toronto, ON, Canada M3K 2C9; Defence Research and Development Canada, Toronto Research Centre, Toronto, ON, Canada M3K 2C9; Faculty of Kinesiology and Physical Education, University of Toronto, Toronto, ON, Canada M5S 2W6; Neurosciences and Mental Health, Hospital for Sick Children Research Institute, Toronto, ON, Canada M5G 0A4; Department of Medical Imaging, University of Toronto, Toronto, ON, Canada M5G 1X8; Department of Diagnostic and Interventional Radiology, Hospital for Sick Children, Toronto, ON, Canada M5G 1X8; Department of Psychology, University of Nottingham, Nottingham NG7 2RD, UK

**Keywords:** delta oscillations, gamma oscillations, neurodegeneration, electrophysiological imaging, tau

## Abstract

Concussion is a public health crisis that results in a complex cascade of neurochemical changes that can have life-changing consequences. Subconcussions are generally considered less serious, but we now realize repetitive subconcussions can lead to serious neurological deficits. Subconcussions are common in contact sports and the military where certain personnel are exposed to repetitive occupational blast overpressure. Post-mortem studies show subconcussion is a better predictor than concussion for chronic traumatic encephalopathy—a progressive and fatal neurodegenerative tauopathy, only diagnosable post-mortem—thus, an in vivo biomarker would be transformative. Magnetoencephalography captures the dynamics of neuronal electrochemical action, and functional MRI shows that functional connectivity is associated with tauopathy patterns. Therefore, both imaging modalities could provide surrogate markers of tauopathy. In this cross-sectional study, we examined the effects of repetitive subconcussion on neuronal activity and functional connectivity using magnetoencephalography and functional MRI, and on neurological symptoms and mental health in a military sample. For magnetoencephalography and outcome analyses, 81 participants were split into ‘high’ and ‘low’ blast exposure groups using the generalized blast exposure value: *n* = 41 high blast (26.4–65.7 years; 4 females) and *n* = 40 low blast (28.0–63.3 years; 8 females). For functional MRI, two high blast male participants without data were excluded: *n* = 39 (29.6–65.7 years). Magnetoencephalography revealed disrupted neuronal activity in participants with a greater history of repetitive subconcussions, including neural slowing (higher delta activity) in right fronto-temporal lobes and subcortical regions (hippocampus, amygdala, caudate, pallidum and thalamus), and functional dysconnectivity in the posterior default mode network (lower connectivity at low and high gamma). These abnormalities were independent of concussion or traumatic stress history, and magnetoencephalography showed functional dysconnectivity not detected in functional MRI. Besides magnetoencephalography changes, those with higher blast exposure had poorer somatic and cognitive outcomes, with no blast-related differences in mental health or associations between neurological symptoms and neuronal activity. This study suggests that repetitive subconcussions have deleterious effects on brain function and that magnetoencephalography provides an avenue for both treatment targets by identifying affected brain regions and in prevention by identifying those at risk of cumulative subconcussive neurotrauma.

## Teaser

Solar *et al*. report that blast-related repetitive subconcussions are associated with neural slowing and functional dysconnectivity in critical brain regions (detected by magnetoencephalography but not functional MRI) and somatic and cognitive impairments, independent of concussion and traumatic stress history—highlighting the potential utility of magnetoencephalography as a marker for neurodegeneration.

## Graphical Abstract

**Graphical Abstract:**
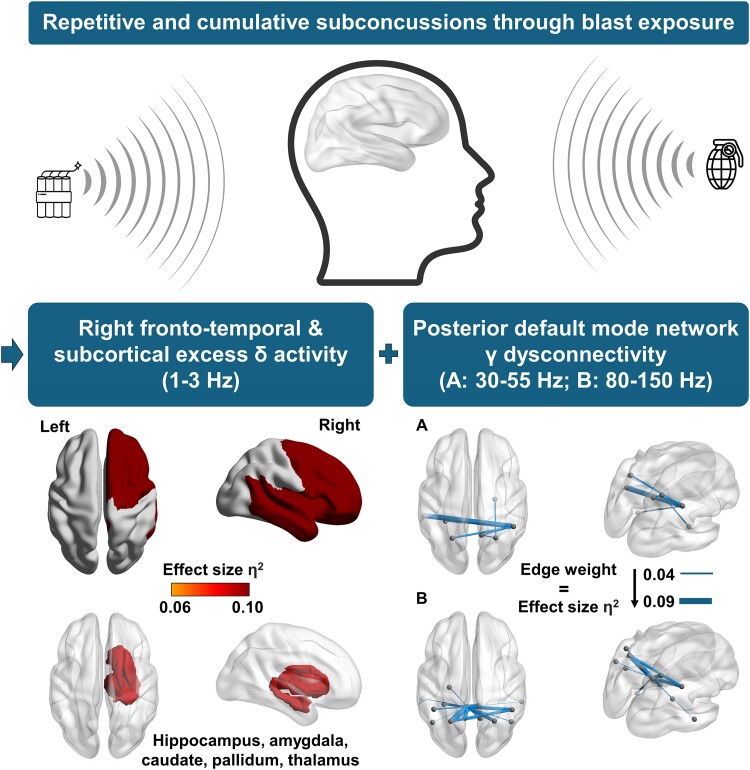

## Introduction

Concussion, or mild traumatic brain injury (mTBI), involves a complex cascade of neurochemical changes and neurological symptoms that can have life-changing consequences.^1^ Repetitive subconcussions were thought to be less serious, and have been largely overlooked up until recently^2-4^ but are now considered deleterious, and occur from repetitive head impacts (RHIs) common in contact sports^5,6^ and in military settings with occupational repetitive blast overpressure (ReBOP) exposure (e.g. explosions, small arms and heavy weapons).^7-11^ Post-mortem studies in athletes reveal that the accumulated duration of play^5,6^ and total force^6^ are better predictors than concussion history for the presence and severity of chronic traumatic encephalopathy (CTE), a progressive and fatal neurodegenerative tauopathy, which is distinct from concussions and persistent post-concussion symptoms (PPCSs). Yet, the pathogenic mechanisms of CTE are not well understood,^12^ although histopathology suggests that expression of glial fibrillary acidic proteins at the cortical grey–white matter interface are a neuropathological hallmark of cumulative mild blast or impact neurotrauma.^13^ However, post-mortem examinations have shown that CTE was not often detected in the brains of military members, as evidenced by modest neuropathologic changes, and CTE risk ratios were higher for decedents who experienced other traumatic brain injuries (TBIs) outside of the military (e.g. contact sports) than those who only had blast exposure or military-related TBI.^14^

Subconcussions have been studied in the context of RHI, but occupational blast exposure provides a model for the effects of cumulative, repetitive subconcussions common in certain military roles.^15^ Military personnel and veterans exhibit neurobehavioural symptoms that can mimic PPCSs, dementia and post-traumatic stress disorder (PTSD). These include somatic, cognitive and emotional symptoms and mental health issues (e.g. anxiety and depression).^7-10^ Also, higher lifetime subconcussive blast exposure is associated with poorer brain-related outcomes, including worse neurobehavioural symptoms (e.g. sleep and fatigue).^16^ Moreover, blast-related military service is included in the National Institute of Neurological Disorders and Stroke diagnostic criteria for traumatic encephalopathy syndrome, the clinical disorder that precedes CTE.^14,17^

Given that CTE and proteinopathic dementias can only be diagnosed post-mortem, an *in vivo* predictive biomarker would be transformative. PET radiotracer binding to tau with flortaucipir offers a potential avenue,^18^ with uptake correlating with blast exposure in frontal, temporal, occipital and cerebellar regions in warfighters and veterans.^11^ However, a limitation of PET includes its high cost and concern of non-specific binding.^19^ A PET-tau study in Alzheimer’s dementia found that tauopathy colocalized with abnormal synaptic function quantified by magnetoencephalography (MEG),^20^ and another investigation in primary four-repeat tauopathies showed that tauopathy patterns were associated with functional connectivity quantified by functional MRI (fMRI).^21^ Together, this research suggests that MEG and fMRI can quantify or identify dysfunction associated with tauopathy at a fraction of the cost of PET.

Electrophysiology with MEG offers a highly sensitive, non-invasive means to image the dynamics of neuronal electrochemical action by measuring magnetic fields generated by primary neuronal currents, allowing for micro- to macro-level examinations of neuronal circuits. Neuronal oscillations are important surrogate measures of cell physiology, indexing the rhythmic activity of brain excitability and inhibition, with abnormalities in the temporal pattern of oscillatory activity indicating pathology.^22,23^ MEG can measure neuronal oscillations because of its exceptional temporal sampling rate, whereas fMRI relies on the blood oxygen level dependent (BOLD) signal, a slower haemodynamic proxy for neural activity.

Pathological rhythmic neuronal activity—or ‘oscillopathies’—and functional connectivity are important pathogenic markers of psychiatric and neurological disorders, including concussion,^24-29^ PTSD,^24^ Parkinson’s disease^30^ and Alzheimer’s disease.^31^ ‘Pathological slowing’ of rhythmic activity and changes in functional connectivity are common features of brain injury and neurodegeneration in military personnel and veterans with concussion.^27^ In neurodegenerative disease specifically, neuronal slowing in Parkinson’s disease has been observed in parieto-occipital cortices^30^ and neuronal slowing in Alzheimer’s disease predicts functional impairment and amyloid burden.^31^

Although there are functional neuroimaging studies of military personnel who have sustained an mTBI from blast exposure,^32-35^ there are no multimodal studies on the impact of repetitive subconcussions, or those dissociating the effects of concussion on brain function.^36^ There is one MEG study in combat veterans that identified reduced functional connectivity in blast-related concussion with PTSD^35^; however, they did not disentangle repetitive subconcussive blast exposure from concussion. An MRI study showed that blast exposure resulted in worse cognitive, emotional and somatic symptoms and smaller cortical volumes in the right superior frontal gyrus.^10^ An fMRI study revealed the effect that a single day of breacher training with acute, multiple blast exposures has on the brain, wherein the researchers gave half of the participants a jugular vein compression collar—those without the collars presented with blast-related hyperconnectivity in the working memory and auditory networks, suggesting that the effects of repetitive subconcussion may be managed by simple protective devices.^37^ In sports, a longitudinal fMRI study showed that high school football players exhibited dysfunctional hyperconnectivity across a single season of play despite not showing any symptoms, suggesting pre-symptomatic brain changes.^38^ In line with these head impact studies, both fMRI and MEG reveal varied changes in functional connectivity, including abnormal hyper- and hypoconnectivity in association with different phases of injury post-concussion (see reviews on fMRI, Dogra *et al.*,^39^ and MEG, Allen *et al.*^26^). These studies highlight the value of functional neuroimaging to identify *in vivo* biomarkers of subconcussive neurotrauma before debilitating symptoms emerge.

The pathological mechanism of repetitive subconcussions—including blast—that might lead to CTE remains largely unknown, but repetitive blast exposure provides a model to investigate pathogenesis and novel markers.^40^ Such a model can inform basic science and provide translatable paths to refine training procedures and improve overall health outcomes for operational readiness.^7-10,16,37,38^ There is evidence that repetitive blast damages the central nervous system and/or the central or peripheral vestibular system due to blast waves entering the ear canal and subsequently causing mechanical reverberations throughout the brain.^41,42^ However, other studies argue that the mechanism extends beyond acceleration–deceleration injuries, due to the effect of blast waves on both air-filled organs and/or organs surrounded by fluid-filled cavities within the body.^43,44^

The purpose of this study was to understand the interaction between lifetime blast exposure estimates, neurobehavioural symptoms, mental health and brain function outcomes in Canadian Armed Forces (CAF) and Royal Canadian Mounted Police (RCMP) personnel and veterans exposed to the continuum of repetitive subconcussions from blast overpressure, controlling for concussion [indicated by the Acute Concussion Evaluation (ACE)^45^] and traumatic stress history [indicated by the Brief Trauma Questionnaire (BTQ)^46^]. The generalized blast exposure value (GBEV) tool^15^ was used to capture blast history—we performed a median split of the cohort into two groups of ‘high’ and ‘low’ blast exposure as described in this other protocol,^47^ to understand the impact and relationships between blast, health outcomes and neural activity. Multimodal studies suggest that proteinopathic burden and neurodegeneration correlate with neuronal slowing and changes in brain communication^20^—therefore, we predict that participants with higher blast load will show neuronal slowing (MEG) and abnormal functional connectivity (MEG and fMRI—both of which have revealed abnormal hyper- and hypoconnectivity in concussion^26,39^) alongside worse neurobehavioural and mental health symptoms.

## Materials and methods

### Participants

Participants were recruited as part of a large, multidimensional, cross-sectional and longitudinal study with a transdiagnostic design to examine military brain and mental health. Methods of recruitment included dissemination of study information directly to military units, as well as health service channels and philanthropic organizations dealing with military and veteran brain health (e.g. Project Enlist, Veterans Affairs Canada and other veteran’s groups). Participants were eligible for this study if they were active CAF/RCMP personnel or a veteran of the CAF/RCMP at the time of study enrolment, had sufficient English language skills and cognitive capacity to participate in the study, had no contradictions to MRI or MEG and had no history of moderate or severe TBI. Participant data collection occurred from September 2020 to May 2023 and, along with all analyses, was conducted at the Hospital for Sick Children in Toronto, ON, Canada.

Initially, 84 total participants were recruited; 3 participants did not complete imaging (MEG or fMRI), and of the 81 remaining, 2 completed the MEG scan but not fMRI. For all MEG and outcome analyses, 81 CAF/RCMP personnel and veterans from various backgrounds, including those from combat and emergency response roles involving weapons use, armoured, engineering, operators, explosive disposal and artillery, were divided into two groups based on blast exposure levels assessed by the GBEV questionnaire: *n* = 41 high blast exposure group (aged 26.4–65.7 years; mean 48.1 ± 9.0 years; 4 females) and *n* = 40 low blast exposure group (aged 28.0–63.3 years; mean 47.8 ± 9.5 years; 8 females). For fMRI analyses, two male participants without fMRI data were removed from the high blast group described above so that: *n* = 39 high blast group (aged 29.6–65.7 years; mean 48.6 ± 8.5 years; 4 females). The GBEV is a tool for standardizing a lifetime of blast exposure.^15^ It quantifies units of blast exposure by querying participants about exposure over a lifetime across five categories of blast type/severity. This study was approved by the Hospital for Sick Children Ethics Board. Written informed consent was obtained from all participants in accordance with the Declaration of Helsinki.

### Neurobehavioural and psychiatric outcomes

All participants completed neurobehavioural and mental health assessments, which included the following: the Generalized Anxiety Disorder 7 (GAD7) screener^48^; Patient Health Questionnaire (PHQ9)^49^; PTSD Checklist Military Version (PCL-M)^50^; and the neurobehavioural symptom evaluation from the Sports Concussion Assessment Tool 5th Edition (SCAT5).^51^ Participants also completed brief screeners related to diagnosed lifetime concussion history, using the ACE,^45^ and defined by Department of Defence/Veterans Affairs guidelines for mTBI as injury resulting in loss of consciousness <30 min, post-traumatic amnesia <24 h and a Glasgow Coma Score of 13 or more. Traumatic stress history was also estimated with the BTQ.^46^

### MEG acquisition and analysis

Resting-state MEG data (eyes open) were acquired on a 151-channel CTF system at the Hospital for Sick Children in Toronto. Participants were scanned in the supine position with fiducial coils attached at the nasion and bilateral preauricular pits for head motion recording. Data sampling rate was 600 Hz, bandpass filtered offline (high-pass, 1 Hz; low-pass, 150 Hz; notch, 60 Hz) and analysed in the FieldTrip toolbox.^52^ Sensor-level time series were mean centred. Cardiac and ocular artefacts were removed using independent component analysis (ICA). Time series data were divided into 10 s epochs, and the maximum number was retained from the 5 min recording based on the following exclusion criteria: (i) head position did deviate >5 mm during a given epoch and (ii) epochs that contained superconducting quantum interference device resets or exceeded a ±2 pT threshold following ICA component rejection.

MEG co-registration was performed using a single-shell head model per participant based on their anatomical T_1_-weighted MRI data normalized to Montreal Neurological Institute (MNI) space. A beamformer was used to resolve time series data from 90 regions of the Automated Anatomical Labelling (AAL) atlas^53^ with the parcel centroid of each AAL region used as the node location for beamformer reconstruction for which the FieldTrip toolbox^52^ linearly constrained minimum variance vector beamformer^54^ was used, with 5% Tikhonov regularization. At each AAL node, lead fields were calculated from the template single-shell head model for a unit current dipole across three dimensions. Beamformer weights of nodal neural activity were calculated through projection of the sensor weights along the dimensional axis with the highest singular value decomposition variance that produces a neural activity time series for each node.

The retained time series data from virtual electrodes were *Z*-scored (i.e. mean centred and variance normalized). These broadband regional time courses per node were then filtered into delta (1–3 Hz), theta (3–7 Hz), alpha (8–14 Hz), beta (15–25 Hz), low gamma 1 (30–55 Hz), low gamma 2 (65–80 Hz) and high gamma (80–150 Hz) ranges. To reduce artificially inflated coupling that can result from beamformer leakage, a symmetric orthogonalization correction procedure was used.^55^ Welch’s method was used to obtain the power spectrum density (PSD) at each AAL node. Lobe-wise average power was calculated per hemisphere for each participant by averaging across each AAL node within a given lobe.^56^ Subsequently, the Hilbert transform was applied to the band-limited time course to generate instantaneous estimates of the amplitude envelope, which was then down-sampled to 1 Hz.^57^ Pearson correlations were calculated between all AAL node pairs to quantify functional coupling. Amplitude envelope coupling (AEC) was chosen over other measures of neural communication (for example phase synchronization) as it is the most reliable measure of connectivity across sessions and over individuals,^24,58,59^ pointing to the greatest replicability as well as the lowest susceptibility to co-registration-related errors.^60^

### MRI acquisition and analysis

MRI data were acquired on a 3T Siemens PrismaFit scanner with a 20-channel head and neck coil at the Hospital for Sick Children in Toronto. For source localization of MEG and fMRI data, 3D magnetization-prepared rapid gradient echo T_1_-weighted images were acquired with 0.8 mm isotropic voxel size in 5:01 min (repetition time, TR, 1870 ms; echo time, TE, 3.1 ms; inversion time, TI, 945 ms; field of view, FOV, 240 × 256 mm with 240 0.8 mm slices).

Resting-state fMRI data (eyes open) were acquired with 3 mm isotropic voxel size in 5 min (TR, 1500 ms; TE, 30 ms), and standard preprocessing was completed with the Data Processing Assistant for Resting-State fMRI toolbox^61^ and the SPM8 toolbox^62^ (http://www.fil.ion.ucl.ac.uk/spm). First, slice time correction was applied to the interleaved sequence acquisition, and then realignment of the volumes was accomplished by calculating a six-parameter rigid body spatial transformation.^63^ None of the participants exhibited >3° rotation or >3 mm displacement in any direction across the acquisition; therefore, no volumes were discarded. Next, normalization to MNI space was performed through unified T_1_-weighted image segmentation. The global signal was filtered out along with nuisance covariates including head motion parameters, white matter (WM) signal and CSF signal to minimize the effects of non-neuronal oscillations and motion.^64-67^ Next, images were bandpass filtered at 0.01–0.1 Hz to retain low-frequency oscillations in the resting-state fMRI, which are believed to represent neuronal activity,^68^ while suppressing low-frequency drifts and high-frequency noise resulting from other physiological activity.^69,70^ BOLD time domain signals were extracted from every voxel of the same 90 AAL atlas regions as MEG.^53^ For each individual AAL region, the mean time series were calculated across a parcel and then used to estimate functional connectivity with Pearson correlations.^71^

### Statistical analysis

Statistical analyses were performed using MATLAB version R2022b, JASP version 0.18.0 (JASP, 2023) and the Network-Based Statistic (NBS) toolbox.^72^ False positives due to multiple comparisons were controlled using the Benjamini–Hochberg false discovery rate method at a significance threshold of *P* < 0.05. There are known effects of sex, age, concussion and psychological trauma in these brain and outcome measures; thus, these confounders were controlled for as described below.

#### Participant demographics and outcomes

Analyses of covariance (ANCOVAs) were used to test for blast-related group differences in outcome measures (GAD7, PHD9, PCL-M and SCAT5) while controlling for age, sex, traumatic stress history (BTQ) and number of diagnosed concussions (ACE). ANCOVA was also used to test for group differences in outcomes, stratified by concussion/no concussion history, while controlling for age and sex.

#### PSD analysis

ANCOVAs were used to test for blast-related group differences in power across all seven frequency bands across hemispheric lobes by calculating the average power for each AAL atlas node in the left and right frontal, temporal, parietal and occipital lobes, and subcortical regions (grouping hippocampus, amygdala, caudate, pallidum and thalamus) while controlling for age, sex, psychological trauma and number of concussions.

#### Network-based analysis

The NBS toolbox was used to test group differences in functional connectivity within eight networks based on AAL atlas nodes [anterior default mode network (aDMN), posterior DMN (pDMN), sensorimotor network (SMN), salience network (SN), memory network (MN), visual network (VN), central executive network (CEN) and attention network (AN)] in the seven MEG frequency bands and single fMRI connectomes while controlling for age, sex, psychological trauma and number of concussions (statistical model: threshold = 3.1; *F*-test; 5000 permutations; NBS method; extent component size).

#### Concussion group analysis

Conveniently, of the 81 total participants, *n* = 38 (aged 26.4–65.7 years; mean 48.6 ± 9.5 years; 5 females) had a history of a clinical and confirmed diagnosis of at least one concussion, and the remaining *n* = 43 (aged 28.0–61.9 years; mean 47.3 ± 8.9 years; 7 females) did not; therefore, we also conducted an exploratory between-group analysis based on concussion history, covarying for age, sex, psychological trauma and blast exposure as quantified by the GBEV, to examine concussion-specific changes in brain function with MEG. For the fMRI analyses, the two male participants without fMRI data were removed: one from the concussion group (*n =* 37; aged 29.6–65.7 years; mean 49.2 ± 8.9 years; 5 females) and another from the non-concussion group (*n =* 42; aged 28.0–61.9 years; mean 47.3 ± 9.0 years; 7 females).

#### Brain function and outcome association analyses

As a *post hoc* analysis, regressions were conducted between any brain measures (MEG power or connectivity, fMRI connectivity) and outcomes (mental health, neurological) that showed significant blast-level group differences while controlling for age, sex, psychological trauma and number of concussions.

## Results

### Demographics

**Figure 1:**
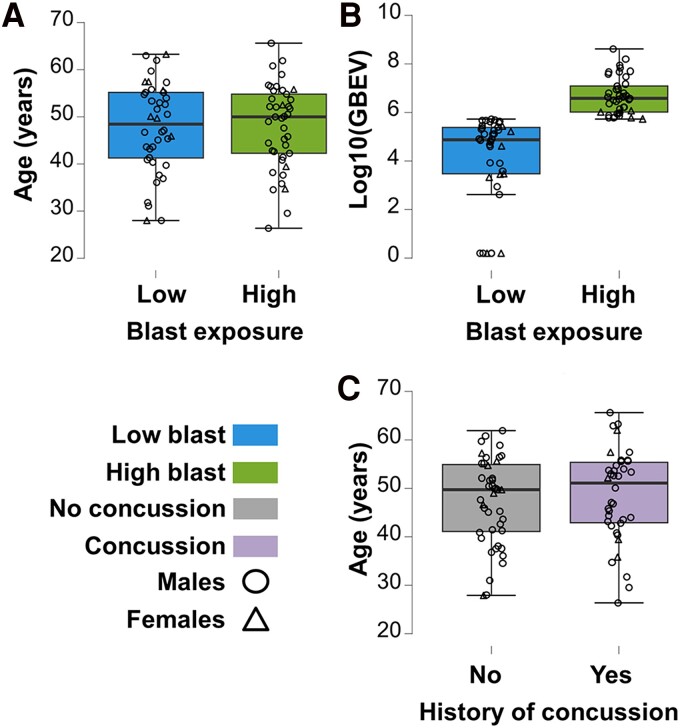
**Demographics.** Age distribution for the entire cohort divided by (**A**) lifetime blast exposure and (**C**) concussion history. (**B**) Log_10_-transformed GBEV plotted per participant, stratified by high and low blast exposure (blue—low blast exposure; green—high blast exposure; grey—no history of concussion; purple—history of at least one concussion; circles—males; triangles—females).

The age of the high blast group (*n* = 41; range: 26.4–65.7 years; mean: 48.1 ± 9.0 years) did not differ from the low blast group (*n* = 40; range: 28.0–63.3 years; mean: 47.8 ± 9.5 years), nor did sex ratios (high blast group: 37 males, 4 females; low blast group: 32 males, 8 females) (Fig. 1A), and there were no age or sex differences when the two high blast male participants were removed for fMRI analyses due to lack of data (*n* = 39; range: 29.6–65.7 years; mean 48.6 ± 8.5 years; 35 males, 4 females). Additionally, when re-stratifying the cohort by concussion history, the age of the concussion group (*n* = 38; range: 26.4–65.7 years; mean: 48.6 ± 9.5 years) did not differ from the non-concussion group (*n* = 43; range: 28.0–61.9 years; mean: 47.3 ± 8.9 years), nor did sex ratios (concussion group: 33 males, 5 females; non-concussion group: 36 males, 7 females), and there were no age or sex differences when the two male participants without fMRI data were removed, including one from the concussion group (*n* = 37; range: 29.6–65.7 years; mean: 49.2 ± 8.9 years; 32 males, 5 females) and another from the non-concussion group (*n* = 42; range: 28.0–61.9 years; mean: 47.3 ± 9.0 years; 35 males, 7 females) (Fig. 1B).

### Neurological symptoms due to repetitive subconcussive overpressure, not concussion history

**Figure 2:**
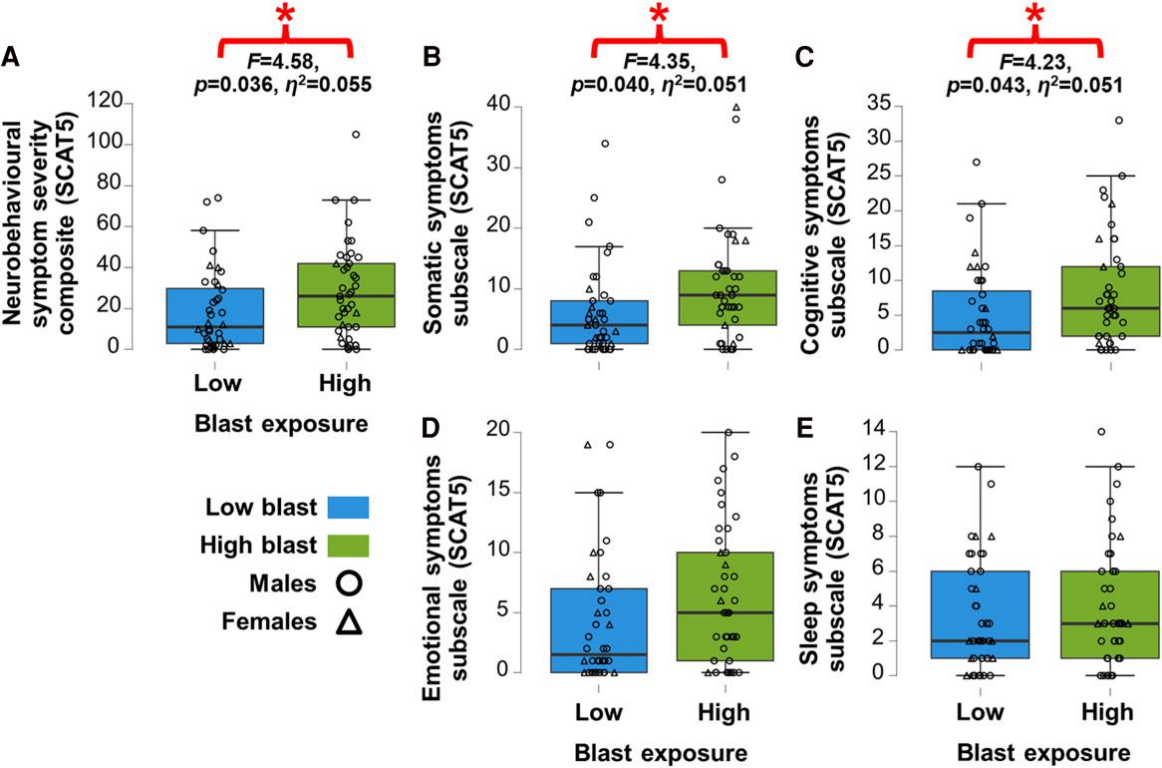
**Neurobehavioural outcomes are worse in those with greater blast exposure, independent of concussions or traumatic stress history.** ANCOVAs with *F*-tests revealed that the (**A**) overall neurobehavioural symptom severity composite score was significantly higher in the high blast exposure group (green) than the low blast group (blue) [*F*(1,75) = 4.58, *P* = 0.036, *η*^2^ = 0.055], driven by significantly worse (**B**) somatic subscale [*F*(1,75) = 4.35, *P* = 0.040, *η*^2^ = 0.051] and (**C**) cognitive subscale symptoms [*F*(1,75) = 4.23, *P* = 0.043, *η*^2^ = 0.051]—with no significant group differences in the subscale measures of (**D**) emotional [*F*(1,75) = 3.79, *P* = 0.055, *η*^2^ = 0.043] or (**E**) sleep symptoms [*F*(1,75) = 0.98, *P* = 0.33, *η*^2^ = 0.012], all indicated by the SCAT5, when controlling for age, sex (circles—males; triangles—females), number of diagnosed concussions and psychological trauma.

The high blast exposure group showed significantly worse overall outcomes on the neurobehavioural symptom severity inventory composite score on the SCAT5 (Fig. 2A), which is based on the four SCAT5 subscale scores that respectively probed somatic, cognitive, emotional and sleep symptoms. Of these subscales, worse overall outcomes for individuals of the high blast group were driven by significantly poorer scores on the SCAT5 subscales of somatic (Fig. 2B) and cognitive symptoms (Fig. 2C), with no blast-related differences in the SCAT5 subscales of emotional (Fig. 2D) or sleep symptoms (Fig. 2E). We controlled for age, sex, psychological trauma history (BTQ) and number of diagnosed concussions (ACE).

**Figure 3:**
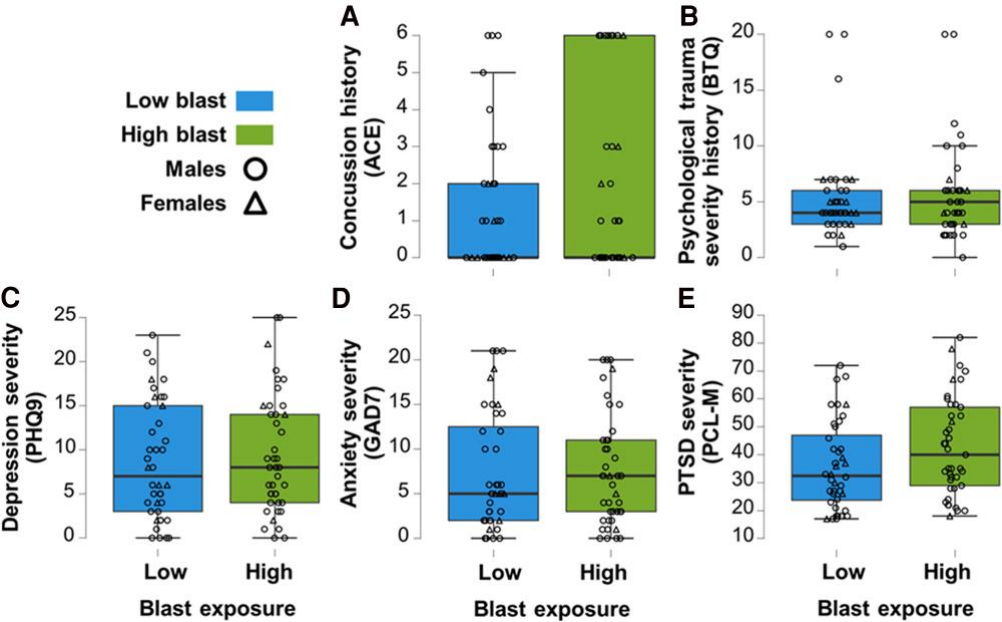
**Concussion history and mental health outcomes did not differ as a function of blast exposure.** ANOVAs with *F*-tests revealed that there were no significant differences between the high blast exposure group (green) and the low blast group (blue) in the (**A**) number of diagnosed concussions [ACE; *F*(1,77) = 1.71, *P* = 0.20, *η*^2^ = 0.021] while controlling for age and sex (circles—males; triangles—females), (**B**) psychological trauma [BTQ; *F*(1,77) = 0.015, *P* = 0.90, *η*^2^ = 1.94E^−4^] while controlling for age, sex and number of diagnosed concussions, nor in mental health outcomes, including (**C**) depression [PHQ9; *F*(1,75) = 0.50, *P* = 0.48, *η*^2^ = 0.006], (**D**) anxiety [GAD7; *F*(1,75) = 0.07, *P* = 0.80, *η*^2^ = 8.20E^−4^] and (**E**) PTSD [PCL-M; *F*(1,75) = 3.68, *P* = 0.059, *η*^2^ = 0.040] severity while controlling for age, sex, number of diagnosed concussions and psychological trauma.

There were no group differences between high and low blast in the number of diagnosed concussions (Fig. 3A), psychological trauma severity (Fig. 3B) and mental health outcomes, including depression (Fig. 3C), anxiety (Fig. 3D) or PTSD (Fig. 3E) symptom severity. Moreover, there were no significant group differences for neurobehavioural, anxiety, depression or PTSD symptoms in the concussion stratified analysis when controlling for age, sex, psychological trauma and blast exposure history. These results suggest that the elevated neurological symptoms experienced by individuals with greater blast exposure are due to repetitive subconcussions and not concussion or traumatic stress history.

### Neural slowing is evident in fronto-temporal and subcortical regions after high blast exposure

**Figure 4:**
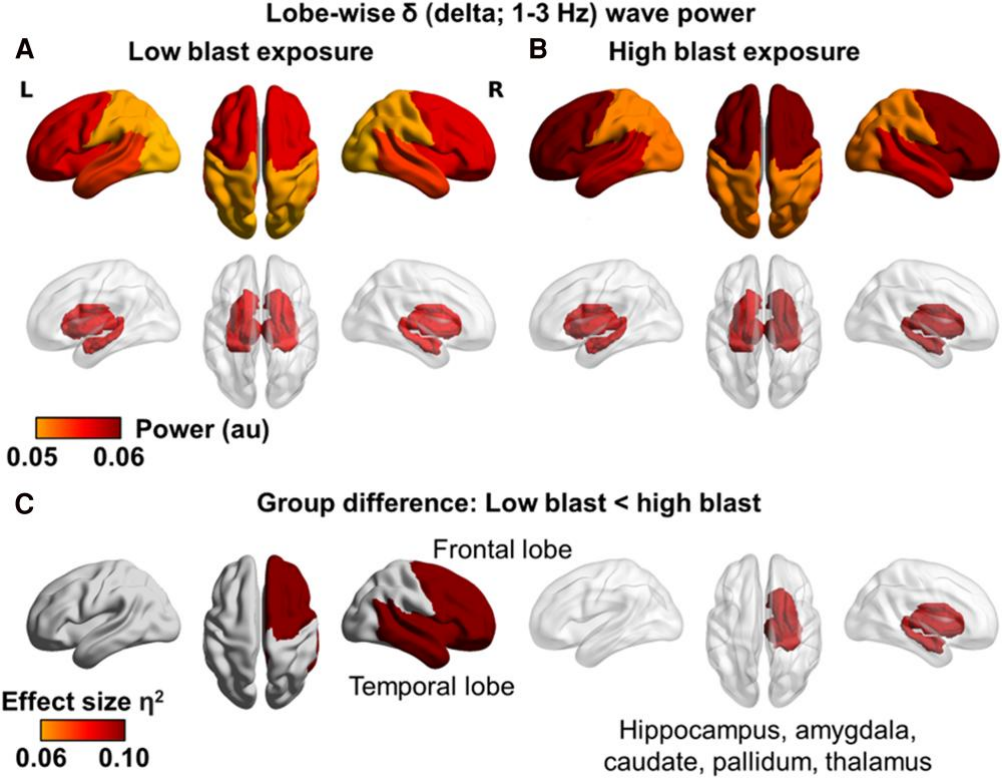
**Repetitive subconcussions are associated with neural slowing in fronto-temporal and subcortical regions, independent of concussion or traumatic stress history.** Lobe-wise delta (1–3 Hz) activity in the (**A**) low and (**B**) high blast exposure groups; ANCOVAs with *F*-tests revealed that (**C**) the high blast exposure group had significantly higher delta power in the right frontal [*F*(1,75) = 8.85, *P* = 0.004, *η*^2^ = 0.10] and temporal [*F*(1,75) = 7.95, *P* = 0.006, *η*^2^ = 0.092] lobes and subcortical regions (hippocampus, amygdala, caudate, pallidum and thalamus) [*F*(1,75) = 7.27, *P* = 0.009, *η*^2^ = 0.084] while controlling for age, sex, number of diagnosed concussions and psychological trauma.

Delta activity (1–3 Hz) was significantly higher in the right frontal and right temporal lobes, as well as in right subcortical regions (grouping seeds in the hippocampus, amygdala, caudate, pallidum and thalamus) in the high blast compared to low blast group when controlling for age, sex, traumatic stress and number of diagnosed concussions (Fig. 4), but not for any other lobes at the delta frequency. There were no group differences for any other of the six frequency bands (see Supplementary Fig. 1 for the lobe-wise power spectrum as a function of blast exposure).

Moreover, there were no group differences when comparing diagnosed concussion versus no concussion history in regional power when controlling for age, sex, trauma and blast exposure. Together, these results suggest that neural slowing in the right fronto-temporal lobes and subcortical regions is due to repetitive subconcussive neurotrauma and not concussion history.

### Decreased pDMN connectivity in high blast exposure as measured by MEG but not fMRI

**Figure 5:**
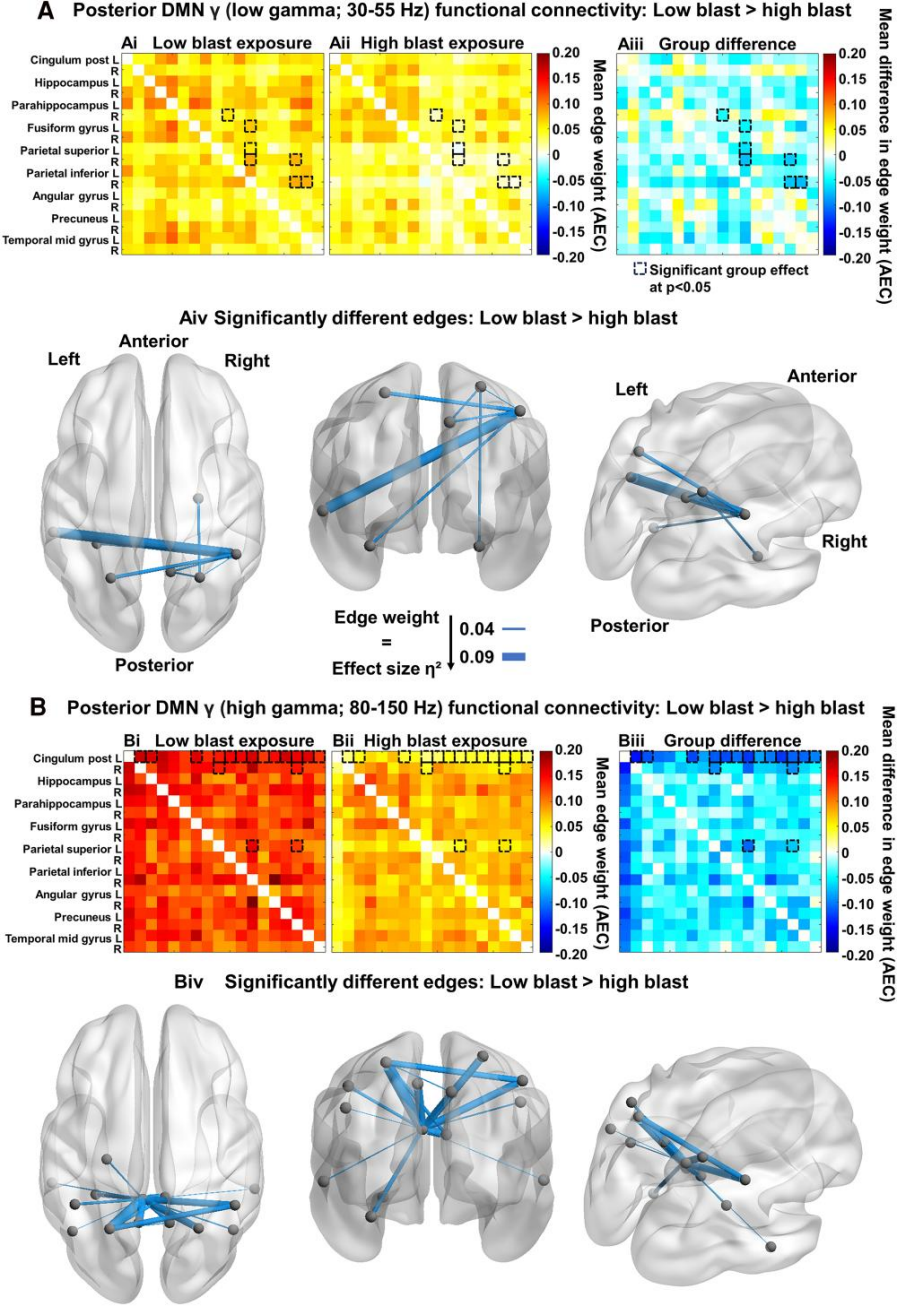
**Repetitive subconcussions are associated with posterior DMN dysconnectivity, independent of concussion or traumatic stress history.** The (**A**) MEG low gamma (30–55 Hz) range and (**B**) high gamma (80–150 Hz) range from the 18 total nodes of the pDMN with (**Ai**, **Bi**) low and (**Aii**, **Bii**) high blast exposure group connectivity matrices scaled by mean edge weight (AEC); NBS analysis with *F*-tests revealed significantly lower connectivity in the high blast relative to the low blast exposure group for 7 nodes and 7 edges at low gamma [**Aiii**, **Aiv**; *F*(1,75) = 4.20, *P* = 0.044, *η*^2^ = 0.053] and 14 nodes and 17 edges at high gamma [**Biii**, **Biv**; *F*(1,75) = 5.31, *P* = 0.024, *η*^2^ = 0.066]. Warm elements in the (**Ai**, **Aii**, **Bi**, **Bii**) group mean matrices indicate higher connectivity, and cool colours indicate lower connectivity; warm elements in the (**Aiii**, **Biii**) group difference matrices (calculated from the low blast group means, **Ai** and **Bi**, subtracted from the high blast group means, **Aii** and **Bii**, respectively) indicate higher connectivity in the high blast group, and cool colours indicate lower connectivity in the high blast group relative to the low blast group. At low gamma, the node with the highest degree was the right inferior parietal (**Aiii**, **Aiv**), and at high gamma, it was the left posterior cingulate (**Biii**, **Biv**).

MEG revealed significant reductions in pDMN functional connectivity for the high blast group at low gamma (30–55 Hz; 7 nodes and 7 edges; Fig. 5A) and high gamma (80–150 Hz; 14 nodes and 17 edges; Fig. 5B).

For low gamma connectivity, the high blast group exhibited dysconnectivity in the pDMN including the right parahippocampus, left fusiform gyrus, bilateral superior parietal cortex, right inferior parietal cortex, right precuneus and left middle temporal cortex (marked with dashed boxes, Fig. 5A)—with no differences in the other seven networks (Supplementary Figs 3 and 4). For high gamma connectivity, the high blast group exhibited dysconnectivity across pDMN areas including the bilateral posterior cingulate cortex (PCC), left hippocampus, left fusiform gyrus, bilateral superior and inferior parietal cortex, bilateral angular gyrus, bilateral precuneus and bilateral middle temporal cortex (marked with dashed boxes, Fig. 5B)—with no differences in the other seven networks (Supplementary Figs 5 and 6).

The regions with the greatest difference (edges scaled by the effect size, Fig. 5A and B) were mainly interhemispheric connections across both gamma ranges in the pDMN. There were no blast exposure group differences in any of the other individual networks at any frequency band, including the AN, CEN, aDMN, MN, SMN, SN and VN (see Supplementary Figs 7 and 8 for MEG delta connectivity across all eight networks, the frequency band in which we identified higher power in the high blast group, and see Supplementary Fig. 2 for MEG low and high gamma power, the frequency bands in which we identified functional dysconnectivity in the high blast group). In contrast to the MEG results, fMRI did not reveal any significant blast-related differences (Supplementary Figs 9 and 10). Additionally, there were no significant group differences in either MEG or fMRI connectivity when stratified by concussion history. These results suggest that (i) the dysconnectivity of the pDMN in the high blast group is due to repetitive subconcussions, and not concussion history, and (ii) MEG is more sensitive to network dysregulation from repetitive subconcussions than fMRI.

### MEG delta power and functional dysconnectivity does not correlate with neurobehavioural outcomes

**Figure 6:**
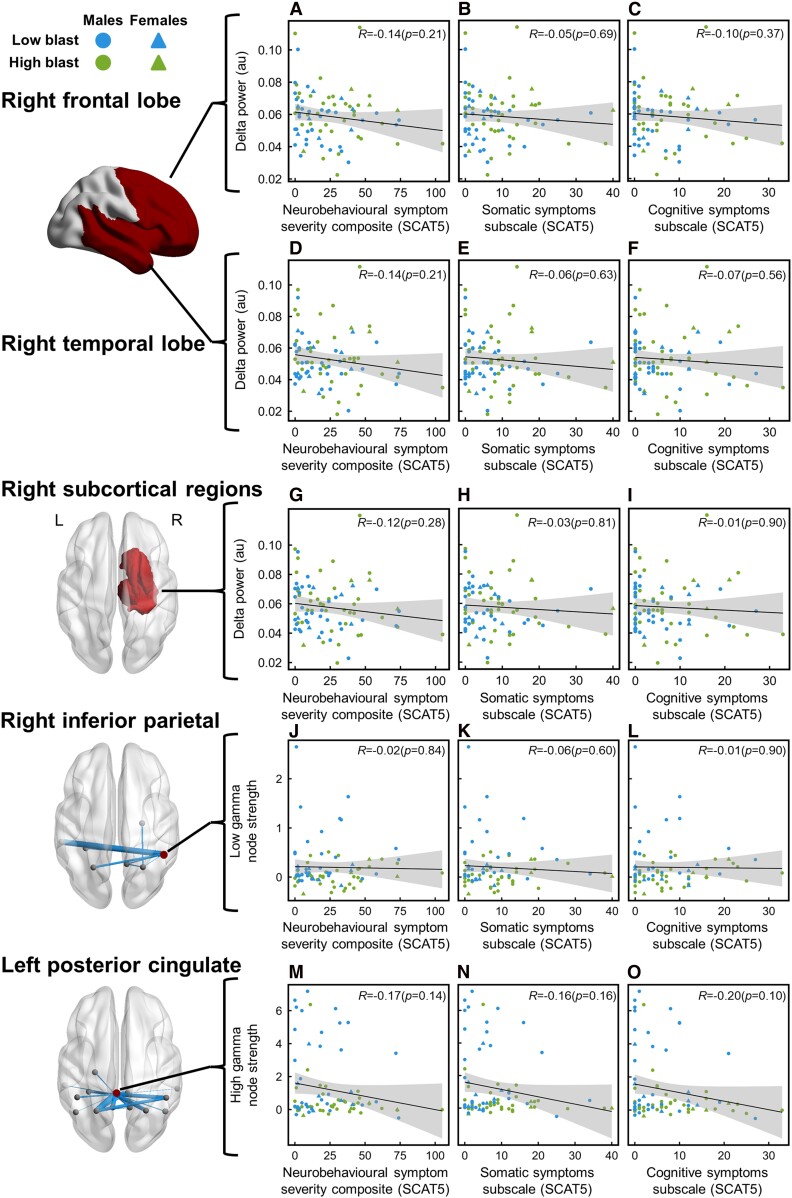
**No blast-related associations between neuronal slowing and dysconnectivity with symptoms.** Partial correlation analysis revealed that there were no significant associations between (**A–I**) neuronal slowing or (**J–O**) dysconnectivity and symptoms (blue—low blast exposure; green—high blast exposure; circles—males; triangles—females; shaded area—95% confidence interval). (**A–C**) Right frontal lobe delta power, (**D–F**) right temporal lobe delta power, (**G–I**) right subcortical regional (hippocampus, amygdala, caudate, pallidum and thalamus) delta power, (**J–L**) right inferior parietal cortex low gamma pDMN connectivity node strength and (**M–O**) left PCC high gamma pDMN connectivity node strength did not significantly correlate with the overall neurobehavioural symptom severity composite score, somatic symptoms subscale or cognitive symptoms subscale (all indicated by the SCAT5).

Despite group differences in delta activity (right frontal and temporal lobes and subcortical regions), pDMN functional connectivity and neurological symptom outcomes (overall neurological symptom severity and subscales of cognitive and somatic symptom severity), there were no significant associations between MEG delta power or functional connectivity and neurological symptom severity when controlling for age, sex, psychological trauma and number of concussions (Fig. 6A–I). For functional connectivity in the pDMN, node strength per participant was calculated for the node with the highest degree of connections with blast-related differences—namely, the right inferior parietal hub at low gamma and the left posterior cingulate hub at high gamma. There were also no significant associations between MEG functional connectivity and neurological symptom severity when controlling for age, sex, psychological trauma and number of concussions (Fig. 6J–O).

## Discussion

### Summary

We present the first multimodal functional neuroimaging evidence of dysregulated neurophysiological functioning in a sample of military members and veterans with a history of repetitive subconcussions, resulting from varying degrees of exposure to blast overpressure—importantly, these results were observed independently of concussion or traumatic stress history. Along with regional slowing of activity, indexed by increased delta activity, and functional dysconnectivity in key brain hubs, those with higher blast exposure also exhibited worse neurological symptoms, especially in the somatic and cognitive domains, with no blast-related differences in mental health outcomes. Despite regional slowing and dysconnectivity and worse neurological symptoms in higher blast exposure, there were no significant associations between MEG power and network functional connectivity and neurological symptom severity.

### Neural slowing in fronto-temporal and subcortical regions

Those with greater blast exposure exhibited neural slowing—elevated delta activity—in fronto-temporal and subcortical regions that was independent of concussion history. Moreover, we observed functional dysconnectivity in the pDMN with MEG (at low and high gamma)—an effect that was absent in our fMRI measurements using the same cohort. Despite group blast-related differences in neurological outcomes and neuronal activity and functional connectivity, there were no associations between neurological outcomes and functional measures.

Animal models show a dose–response curve of blast exposure frequency with tauopathy^73^ that results in abnormal neuronal activity and neurobehavioural deficits.^73-80^ This work is consistent with our findings that show worse cognitive, somatic and physical symptoms, neural slowing in the right fronto-temporal and subcortical regions and lower functional connectivity in the pDMN in those with greater subconcussive load. Rodent studies have shown that even a single blast (i.e. mild–moderate shockwave) can have insidious effects, resulting in poorer memory function and elevated markers of neurodegeneration, microglia activation and gliosis with decreased mature neurons in the prefrontal cortex and limbic areas, including the hippocampus, at 3 months post-exposure—suggesting these areas are particularly susceptible to the effects of blast.^80^

Another rodent study reported brain-wide and hippocampal CTE-linked neuropathology, including myelinated axonopathy, chronic neuroinflammation and neurodegeneration 2 weeks following a single blast, and at 1 month, cognitive impairment, reduced synaptic plasticity and slowed axonal conduction velocity^74^—mechanistically, this would explain our observations of neural slowing. Importantly, the neurobiological mechanism of neuronal slowing differs between acute brain injury and neurodegenerative disease processes: slowing in brain injury is putatively caused by axonal shearing at the grey–white matter boundary,^81^ whereas in neurodegeneration, slowing is caused by proteinopathy, such as microtubule degradation, hyperphosphorylation of tau and resultant neurofibrillary tangles (NFTs).^82,83^ For example, in both civilian and military mTBI, multimodal neuroimaging with MEG detected neuronal slowing and diffusion tensor imaging (DTI) found proximal axonal/WM damage.^84^

Our data suggest that delta oscillations, a stable *in vivo* biomarker of neural slowing, could have clinical utility in a broad range of neurodegenerative diseases, including Parkinson’s disease,^30^ Alzheimer’s disease^31^ and other dementias such as fronto-temporal and vascular dementia.^85^ A study by Goldstein *et al*.^74^ reported CTE-linked cortical and hippocampal neuropathology; it included a complementary *ex vivo* analysis of brains from blast-exposed military veterans, American football athletes and typical controls that revealed CTE-linked tauopathy as identified by tau-immunoreactive NFTs in the frontal, parietal and temporal cortices.^74^ Together, these findings suggest that blast exposure is associated with neurodegeneration that leads to abnormal neuronal activity, providing a potentially viable non-invasive biomarker of disease states.

### High-frequency mediated DMN dysconnectivity

We observed high-frequency pDMN dysconnectivity converging on the PCC. The PCC is well established as a multimodal, polysensory, rich club node, with a metabolic rate on average ∼40% higher than the rest of the brain.^86^ Prior studies have shown PCC dysfunction in numerous acquired brain injury and neurodegenerative states, with PCC lesions severely impacting dynamical functional network configurations associated with general cognitive performance.^87,88^ Patients with mTBI have shown reduced DMN connectivity involving the posterior cingulate,^89^ and interhemispheric posterior DMN functional connectivity is predictive of the level of consciousness in TBI.^90^ Moreover, a longitudinal pre-/post-season fMRI study by DeSimone *et al*.^91^ reported that the number of subconcussive RHIs was key in causing reduced DMN functional connectivity (which was not present pre-season) in a group of football players in the absence of concussion after only a single season of play relative to control athletes from non-contact sports, particularly including dysconnectivity with the left PCC. In our study, we found reduced posterior DMN functional connectivity in the gamma band, gamma being a mechanism thought to selectively and flexibly couple proximal or distant cortical regions to temporally organize neuronal activity through the action of interneurons.^92^

Animal models of tauopathy suggest that tau protein propagation in the brain is promoted by neuronal activity.^93-95^ A recent study of patients with participants along the Alzheimer’s disease spectrum (preclinical, mild cognitive impairment and dementia) indicated that tau (identified with PET) spread along functionally connected (identified by MEG) brain regions.^96^ Another Alzheimer’s disease study in amyloid-positive patients showed lower functional connectivity (identified by fMRI) in the DMN for those with elevated tau (identified with PET) but higher functional connectivity for those with lower tau.^97^ Along this line of work, a study by García-Colomo *et al*.^98^ reported longitudinal increases in functional connectivity (identified by MEG) in correlation with tau (identified with PET) in the precuneus in individuals with a family history of Alzheimer’s disease relative to those without—a brain region that was also identified here as part of the disconnected pDMN. This work suggests that changes in functional connectivity as identified by MEG can be indicative of tauopathy, in turn allowing us to not only track disease progression but also to pre-emptively predict where tauopathy will spread based on functional connectivity. MEG could be utilized in the treatment and diagnosis of brain injury by identifying regions of the brain in affected individuals to deliver precision treatment to reduce further spreading and predict individuals at risk and intervene before tauopathy spreads.

Furthermore, although we identified functional dysconnectivity (interregional desynchrony) at the gamma frequency band (low, 30–55 Hz; high, 80–150 Hz), increased activity (localized synchrony) was observed in the delta range (1–3 Hz). While not explicitly examined here, this cross-spectral and multiscale (local versus long range synchrony) may be related to a phenomenon called cross-frequency coupling (CFC) wherein high-frequency oscillations are modulated by slower oscillations to support communication across different brain regions and integrate information across multiple time scales.^99,100^ MEG studies have shown that concussion is associated with the reorganization of frequency coupling modes and altered CFC.^101,102^ Our present findings suggest future studies examine CFC to further understand the mechanisms of brain injury in relation to subconcussion.

### Association with symptoms

Although worse self-reported neurological symptoms were present with greater blast exposure, there were no significant direct associations between neuronal slowing or dysconnectivity and symptoms. However, neuronal slowing in fronto-temporal and subcortical regions is likely pathogenic in terms of neurobehavioural deficits. The frontal lobe is involved in executive functioning including mental flexibility, goal-directed behaviours, language, learning and memory, and related processes like attention.^103^ The hippocampus—part of our subcortical analysis—is involved in multiple aspects of memory, including visual and auditory working and episodic memory,^104-107^ as well as a breadth of other processes including executive functioning, attention, social behaviours, spatial navigation and language.^107,108^ Together, the self-reported cognitive difficulties align with the regional dysfunction we observed. We believe that additional research is needed to explore more fully the association between MEG network functional connectivity and neurological symptom severity, perhaps based on a more comprehensive set of objective outcome measures (rather than subjective self-report) known to be impacted by ReBOP.

### Translational use

Notably, MEG but not fMRI revealed pDMN functional dysconnectivity in greater blast exposure, suggesting increased sensitivity to detect network-level effects—these effects were in the gamma range, high-frequency oscillatory activity to which fMRI is blind. Gamma activity reflects the coordinated action of neural excitability and inhibition, driven by local microcircuits involving excitatory pyramidal neurons and inhibitory interneurons, described as the pyramidal-interneuron network gamma or interneuron network gamma models.^109-111^

The application of MEG as a potential surrogate marker for neurodegenerative states would inform translatable paths to procedural and institutional change to reduce the deleterious effects of blast exposure in the military and in contact sports. Biomarkers that can identify individuals at risk of neurodegeneration would be critical in preventing further brain injury and can play a role in preventative medicine. Along with other safety measures—translated from rodent models that suggest head immobilization during blast exposure can reduce blast-related brain dysfunction^74^ and that jugular compression during breacher training provides preventative utility^37^—timely integration of longitudinal monitoring with brain-based biomarkers can improve the operational readiness of warfighters and extend the healthy working lifespan of military members and veterans.

### Limitations and future directions

Limitations of this study include the use of a subjective self-reported probe of lifetime blast exposure with the GBEV scale; of course, objective blast exposure data—such as lifetime pressure gauge use—would be advantageous, but due to practical limitations, these data are impossible to capture in our current cohort. Second, multimodal MEG and PET imaging data with flortaucipir could confirm the presence of tau deposition and spatially colocalized neuronal slowing. Confirmatory tau-slowing associations in a single cohort with suspected CTE could reveal mechanistic relationships between tau-induced neurodegeneration and neural slowing, and confirm the latter as a robust surrogate marker for tauopathy. Third, there were no significant relationships between brain measures and self-reported outcomes—a longitudinal approach, with baseline capture before explosives weapons training, would be crucial in establishing slowing as a marker of neurological functioning, given the between-subject heterogeneity and within-subject stability of brain markers, as well as using objective clinical measures (e.g. neuropsychological testing).^56^ Fourth, we did not collect data on other mechanisms of subconcussion; to disentangle specific effects of different types of subconcussion, it is important to also consider non-blast-related subconcussive neurotrauma that may occur in a military setting, such as head impacts from parachute jumps (e.g. Chen *et al*.^112^), and outside a military setting, such as contact sports participation, which can contribute to poorer outcomes.^14^

In future work, we plan to examine WM microstructure in this same cohort and establish whether WM damage leads to neural desynchrony, as WM damage has been reported in contact sport players (football) in the absence of concussions after only a single season of play,^113,114^ as well as MEG tasks in cognitive control, mental flexibility and memory. Furthermore, there has been major, recent interest in expanding MEG analyses beyond canonical frequency band, periodic analyses as applied here and quantifying the aperiodic component of the neurophysiological signal recorded by MEG.^115^ Studies have shown age-related changes in the aperiodic component^116^ and aperiodic alterations in Alzheimer’s disease^117^ and Parkinson’s disease and dementia with Lewy bodies.^118^ Future research should also pair MEG with PET-tau and PET-amyloid as seen in the tauopathy studies mentioned above (e.g. García-Colomo *et al*.^98^; Schoonhoven *et al*.^96^; Schultz *et al*.^97^). Additionally, MEG should also be utilized alongside other biomarkers such as CSF tau and β-amyloid levels,^119^ blood biomarkers of RHIs,^120^ some of which could be indicative of other mechanisms of brain injury in subconcussion including vascular changes,^121-123^ inflammation^121^ and oxidative stress,^122^ and even DNA damage that has been shown to play a potential role in the clinical outcomes associated with post-concussive symptoms.^124^

## Conclusion

Electrophysiological imaging using MEG produced the first evidence of dysregulated neuronal functioning in a cohort with a history of repetitive subconcussions, including military members and veterans with varying degrees of exposure to ReBOP. Notably, these aberrations were observed independently of concussion or traumatic stress history. In addition to regional slowing of activity as indicated by increased delta activity and functional dysconnectivity in key brain hubs and networks, those with worse blast exposure also exhibited worse neurological symptoms, especially in the somatic and cognitive domains, with no blast-related differences in mental health outcomes. MEG has potential utility as a marker for neurodegeneration that can translate to procedural and institutional improvements to minimize harm from subconcussions in military blast exposure and in contact sports.

## Supplementary Material

fcae348_Supplementary_Data

## Data Availability

Defence considerations related to the confidential nature of the human data collected mean it is not publicly available.
