# Supplementary material for: Repetitive subconcussion results in disrupted neural activity independent of concussion history
Source: Brain Commun. 2024 Oct 8;6(5):fcae348. doi: 10.1093/braincomms/fcae348 (PMC11495223; doi:10.1093/braincomms/fcae348)
Supplement: fcae348_Supplementary_Data [file fcae348_supplementary_data.docx]

# Supplementary material


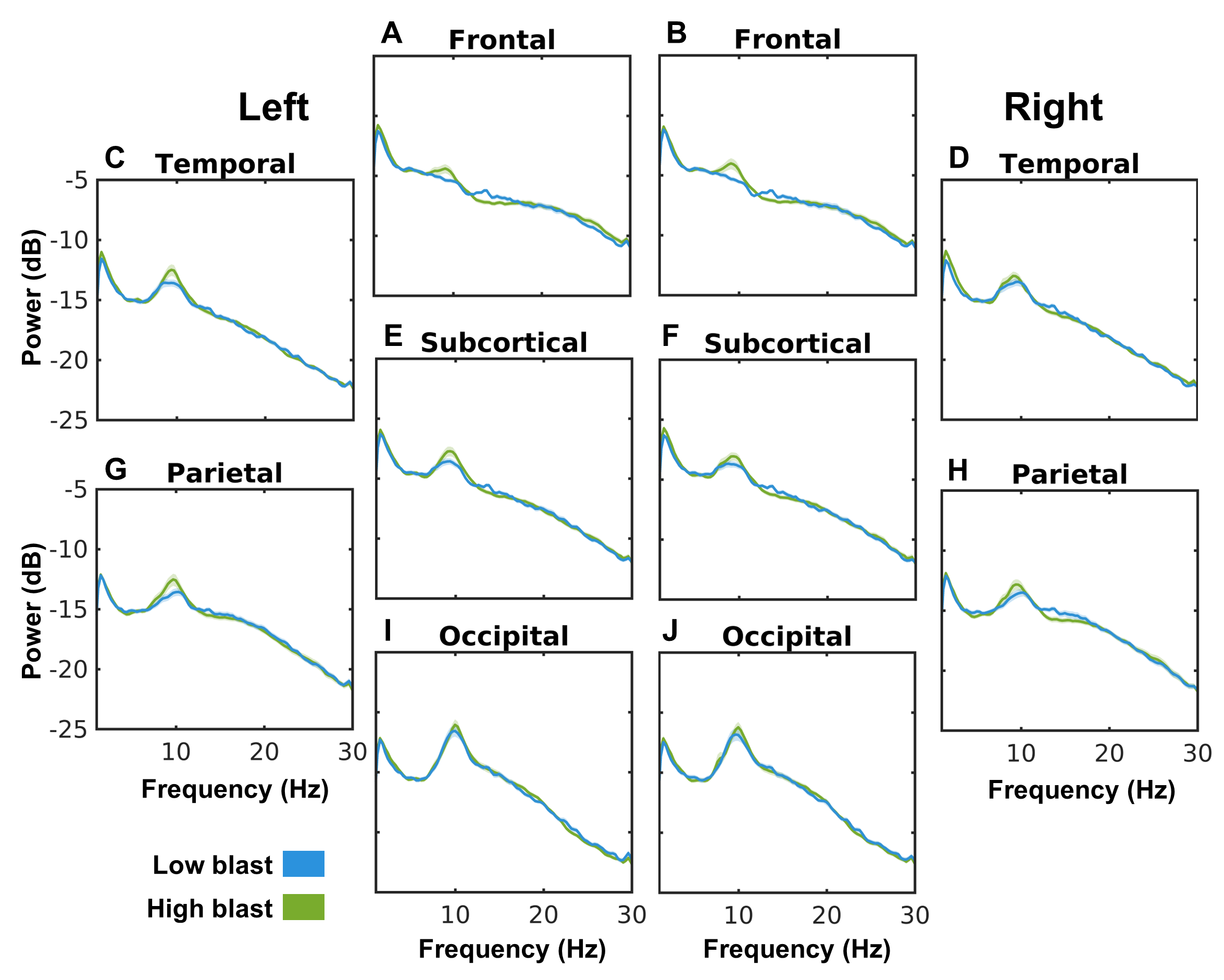


**Supplementary Figure 1:** **Lobe-wise power spectrum as a function of blast exposure.** (**A-J**) Lobe-wise power spectrum for low (blue) and high (green) blast exposure groups for the 1-30 Hz range. (**E**, **F**) Subcortical regions include the hippocampus, amygdala, caudate, pallidum, and thalamus.


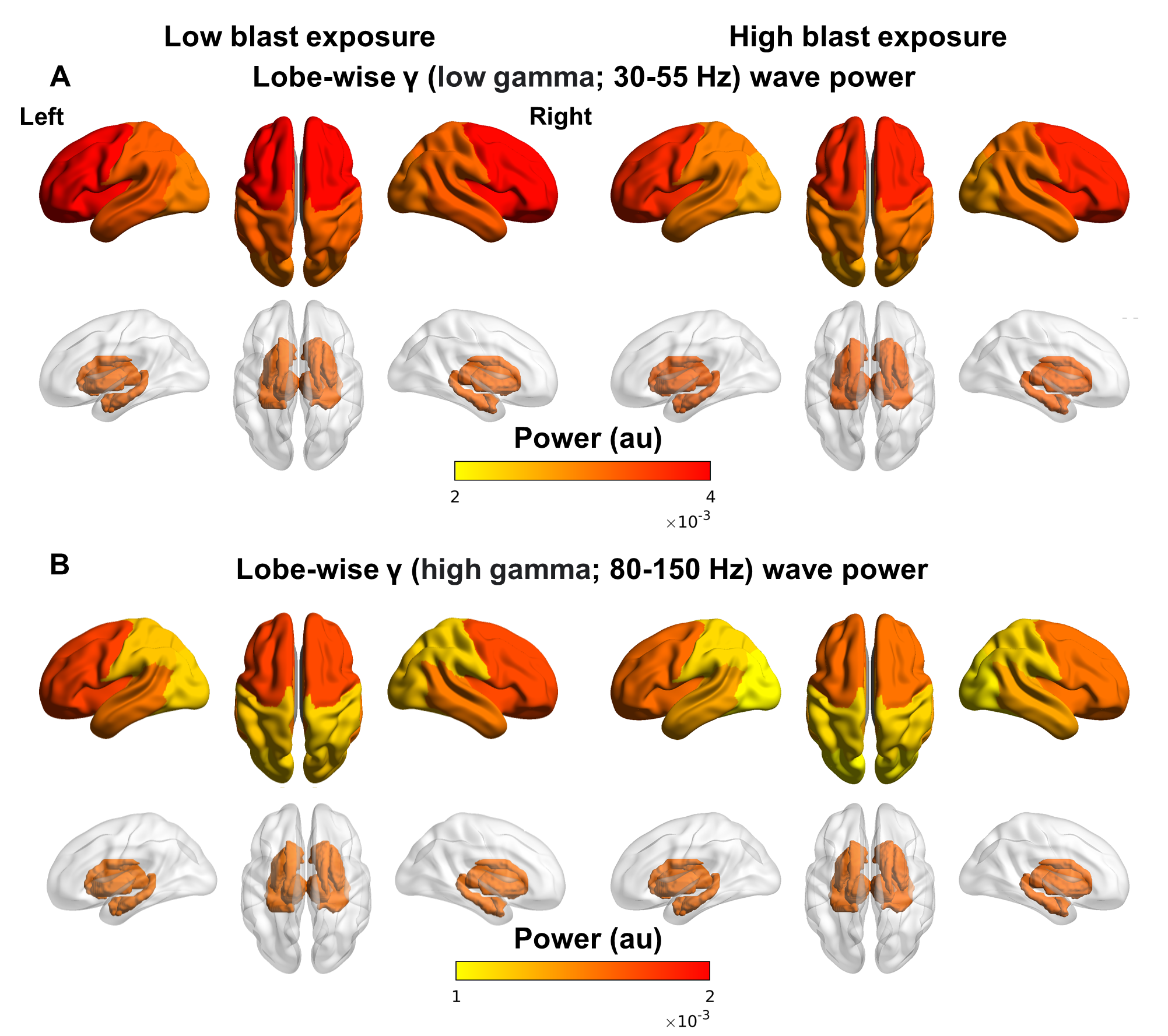


**Supplementary Figure 2.** Lobe-wise (**A**) low gamma (30-55 Hz) and (**B**) high gamma (80-150 Hz) activity in the low and high blast exposure groups. ANCOVAs with F-tests revealed no group differences.


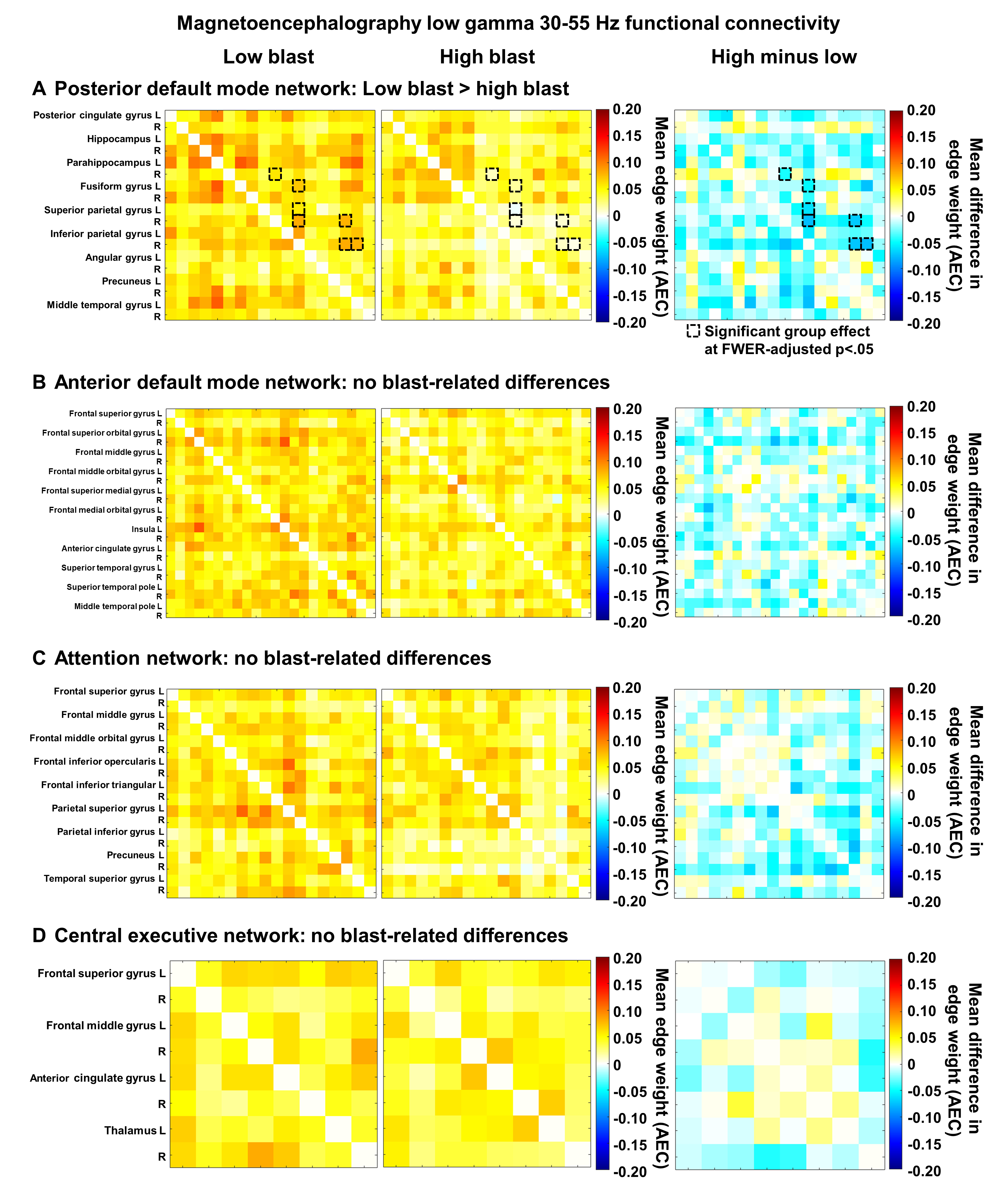


**Supplementary Figure 3:** Functional connectivity matrices scaled by mean edge weight (AEC) measured by resting-state magnetoencephalography (MEG) at the low gamma (30-55 Hz) range in the (**A**) posterior (pDMN) and (**B**) anterior default mode networks (aDMN), (**C**) attention network (AN), and (**D**) central executive control network (CEN) with the mean connectivity for individuals with (**first column**) low blast exposure and (**second column**) high blast exposure, and (**third column**) the group difference. Network-based statistic analyses with F-tests revealed that in the (**A**) pDMN, there was significantly lower connectivity in the high blast relative to the low blast exposure group for 7 nodes and 7 edges at low gamma; the node with the highest degree was the right inferior parietal. There were no blast-related differences in (**B**) aDMN, (**C**) AN, or (**D**) CEN functional connectivity as measured by MEG at low gamma. Warm elements in the high and low blast group mean matrices indicate higher connectivity and cool colours indicate lower connectivity; warm elements in the high minus low blast group difference matrices indicate higher connectivity in the high blast group and cool colours indicate lower connectivity in the high blast group relative to the low blast group.


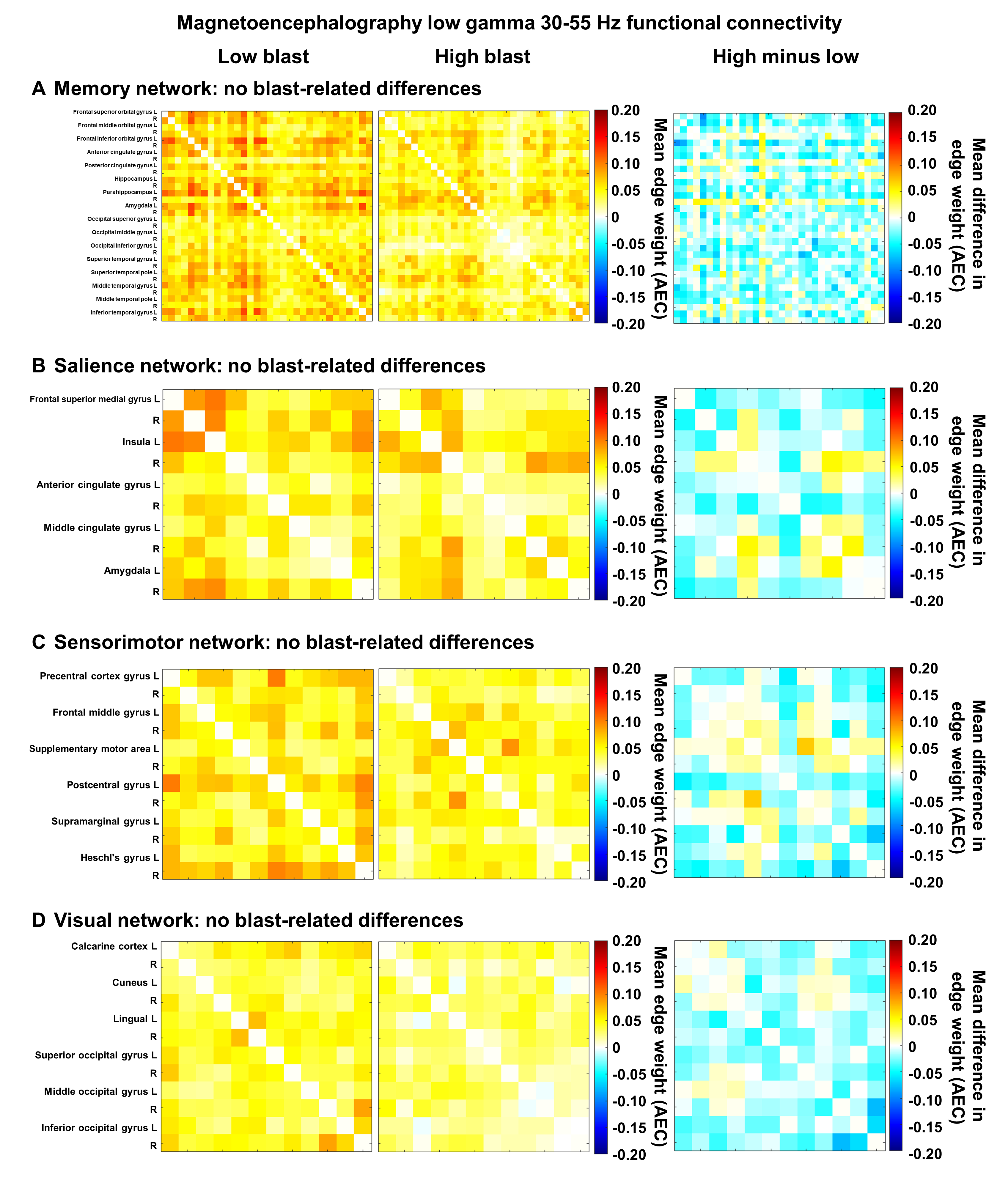


**Supplementary Figure 4:** Functional connectivity matrices scaled by mean edge weight (AEC) measured by resting-state magnetoencephalography (MEG) at the low gamma (30-55 Hz) range in the (**A**) memory network (MN), (**B**) salience network (SN), (**C**) sensorimotor network (SMN), and (**D**) visual network (VN) with the mean connectivity for individuals with (**first column**) low blast exposure and (**second column**) high blast exposure, and (**third column**) the group difference. There were no blast-related differences in (**A**) MN, (**B**) SN, (**C**) SMN, or (**D**) VN functional connectivity as measured by MEG at low gamma. Warm elements in the high and low blast group mean matrices indicate higher connectivity and cool colours indicate lower connectivity; warm elements in the high minus low blast group difference matrices indicate higher connectivity in the high blast group and cool colours indicate lower connectivity in the high blast group relative to the low blast group.


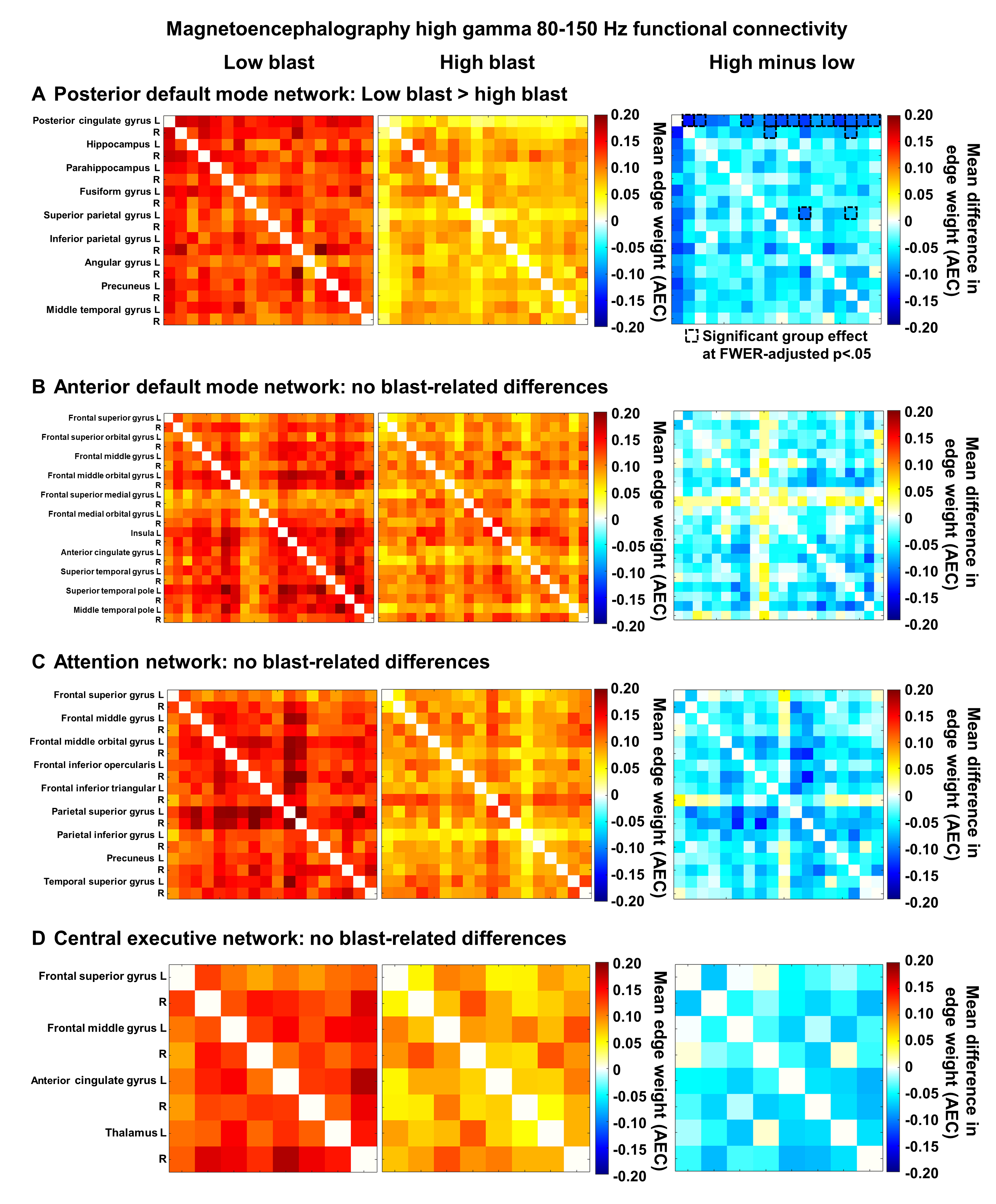


**Supplementary Figure 5:** Functional connectivity matrices scaled by mean edge weight (AEC) measured by resting-state magnetoencephalography (MEG) at the high gamma (80-150 Hz) range in the (**A**) posterior (pDMN) and (**B**) anterior default mode networks (aDMN), (**C**) attention network (AN), and (**D**) central executive control network (CEN) with the mean connectivity for individuals with (**first column**) low blast exposure and (**second column**) high blast exposure, and (**third column**) the group difference. Network-based statistic analyses with F-tests revealed that in the (**A**) pDMN, there was significantly lower connectivity in the high blast relative to the low blast exposure group for 14 nodes and 17 edges at high gamma; the node with the highest degree was the left posterior cingulate. There were no blast-related differences in (**B**) aDMN, (**C**) AN, or (**D**) CEN functional connectivity as measured by MEG at high gamma. Warm elements in the high and low blast group mean matrices indicate higher connectivity and cool colours indicate lower connectivity; warm elements in the high minus low blast group difference matrices indicate higher connectivity in the high blast group and cool colours indicate lower connectivity in the high blast group relative to the low blast group.


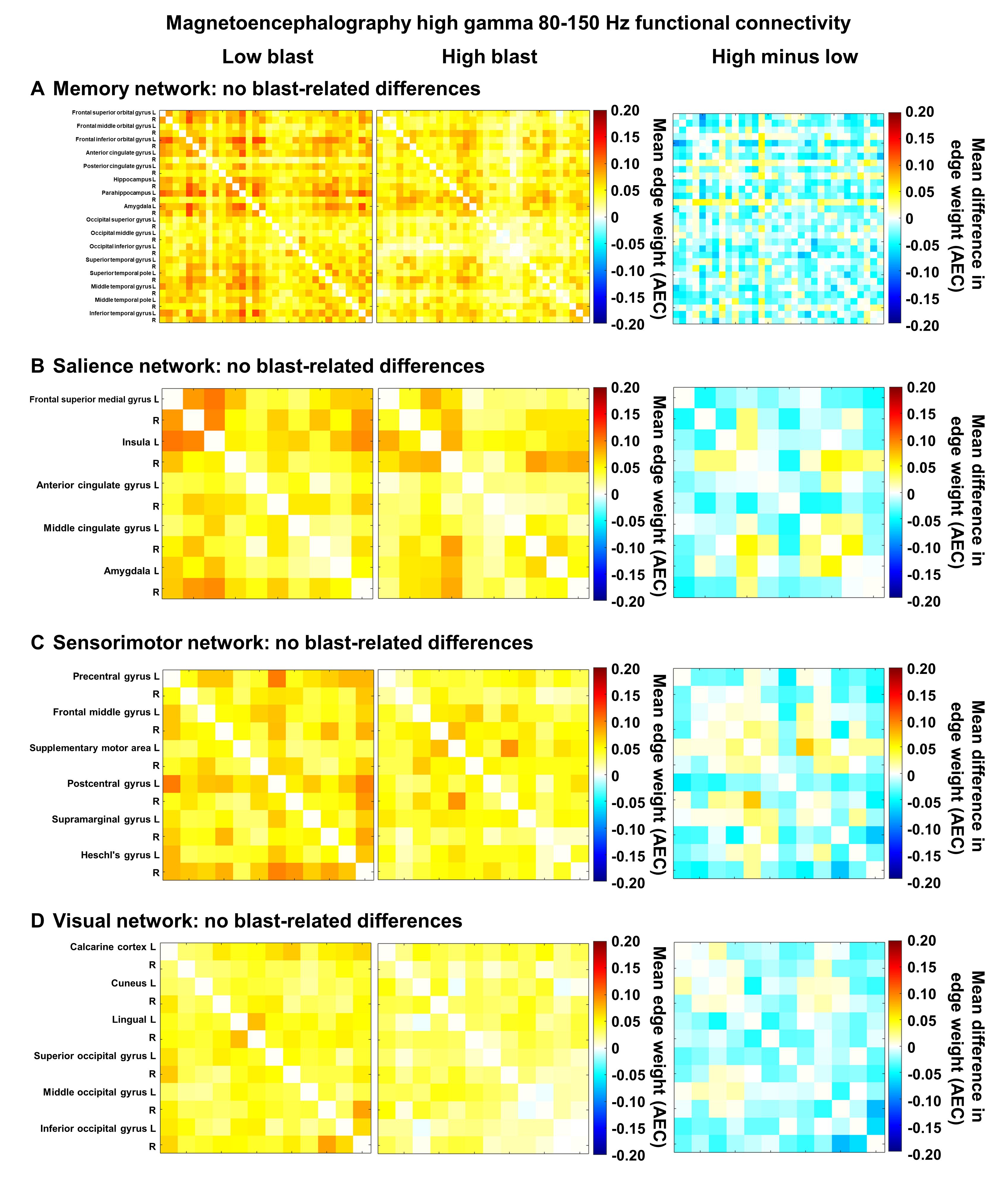


**Supplementary Figure 6:** Functional connectivity matrices scaled by mean edge weight (AEC) measured by resting-state magnetoencephalography (MEG) at the high gamma (80-150 Hz) range in the (**A**) memory network (MN), (**B**) salience network (SN), (**C**) sensorimotor network (SMN), and (**D**) visual network (VN) with the mean connectivity for individuals with (**first** **column**) low blast exposure and (**second** **column**) high blast exposure, and (**third** **column**) the group difference. Network-based statistic analyses with F-tests revealed that there were no blast-related differences in (**A**) MN, (**B**) SN, (**C**) SMN, or (**D**) VN functional connectivity as measured by MEG at high gamma. Warm elements in the high and low blast group mean matrices indicate higher connectivity and cool colours indicate lower connectivity; warm elements in the high minus low blast group difference matrices indicate higher connectivity in the high blast group and cool colours indicate lower connectivity in the high blast group relative to the low blast group.


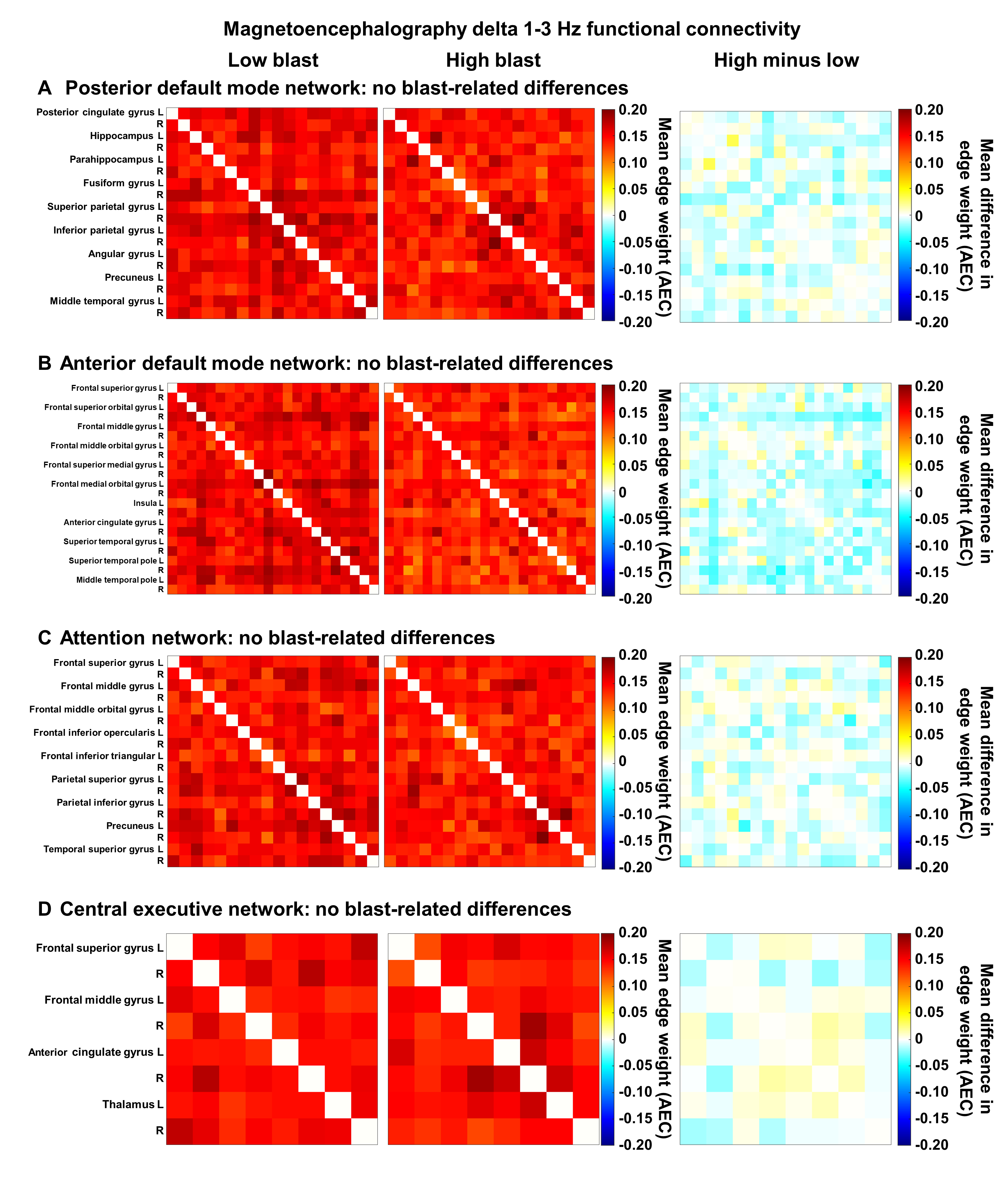


**Supplementary Figure 7:** Functional connectivity matrices scaled by mean edge weight (AEC) measured by resting-state magnetoencephalography (MEG) at delta (1-3 Hz) range in the (**A**) posterior (pDMN) and (**B**) anterior default mode networks (aDMN), (**C**) attention network (AN), and (**D**) central executive control network (CEN) with the mean connectivity for individuals with (**first** **column**) low blast exposure and (**second** **column**) high blast exposure, and (**third** **column**) the group difference. Network-based statistic analyses with F-tests revealed no blast-related differences in (**A**) pDMN, (**B**) aDMN, (**C**) AN, or (**D**) CEN functional connectivity as measured by MEG at delta. Warm elements in the high and low blast group mean matrices indicate higher connectivity and cool colours indicate lower connectivity; warm elements in the high minus low blast group difference matrices indicate higher connectivity in the high blast group and cool colours indicate lower connectivity in the high blast group relative to the low blast group.


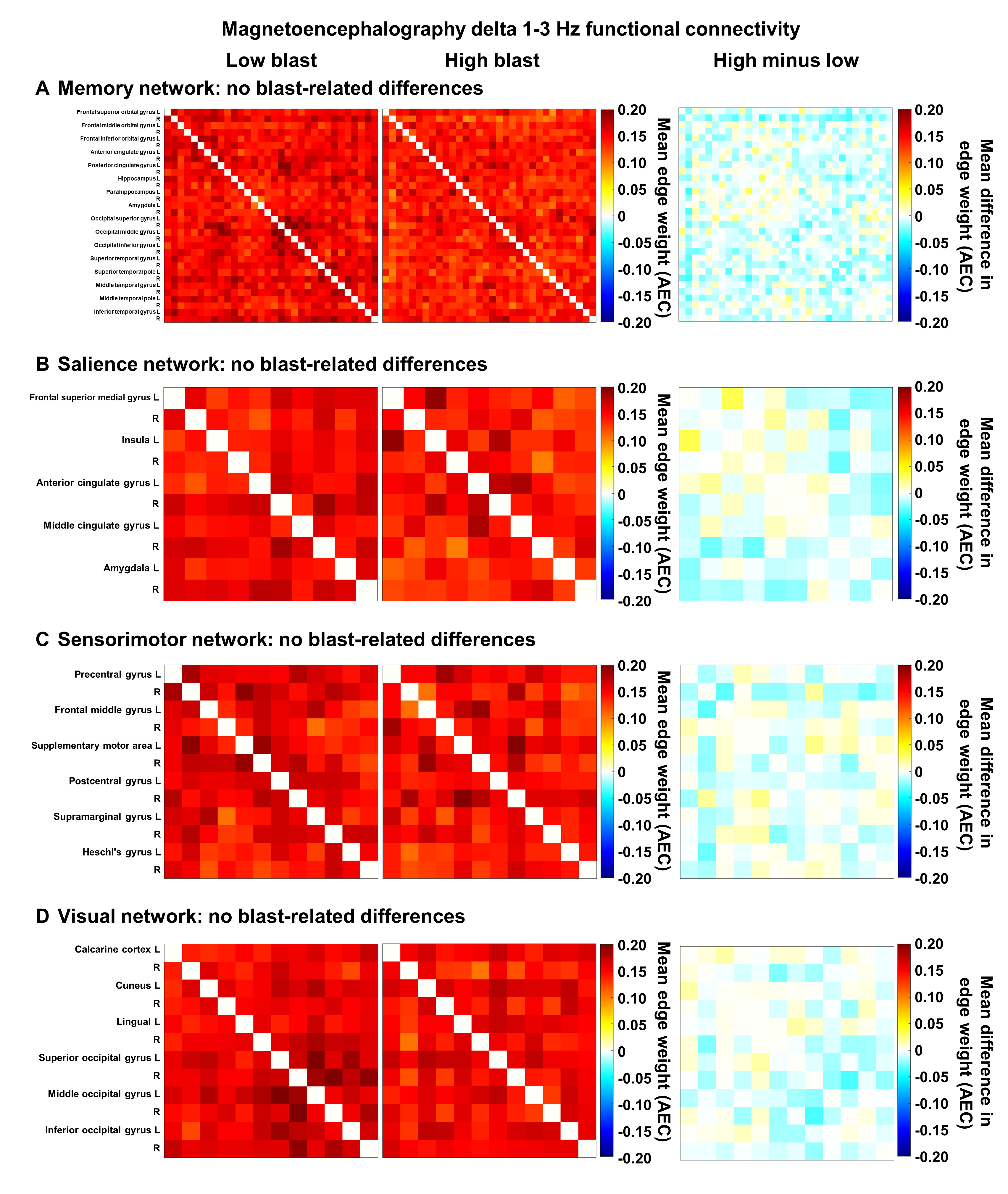


**Supplementary Figure 8:** Functional connectivity matrices scaled by mean edge weight (AEC) measured by resting-state magnetoencephalography (MEG) at delta (1-3 Hz) range in the (**A**) memory network (MN), (**B**) salience network (SN), (**C**) sensorimotor network (SMN), and (**D**) visual network (VN) with the mean connectivity for individuals with (**first** **column**) low blast exposure and (**second** **column**) high blast exposure, and (**third** **column**) the group difference. Network-based statistic analyses with F-tests revealed no blast-related differences in (**A**) MN, (**B**) SN, (**C**) SMN, or (**D**) VN functional connectivity as measured by MEG at delta. Warm elements in the high and low blast group mean matrices indicate higher connectivity and cool colours indicate lower connectivity; warm elements in the high minus low blast group difference matrices indicate higher connectivity in the high blast group and cool colours indicate lower connectivity in the high blast group relative to the low blast group.


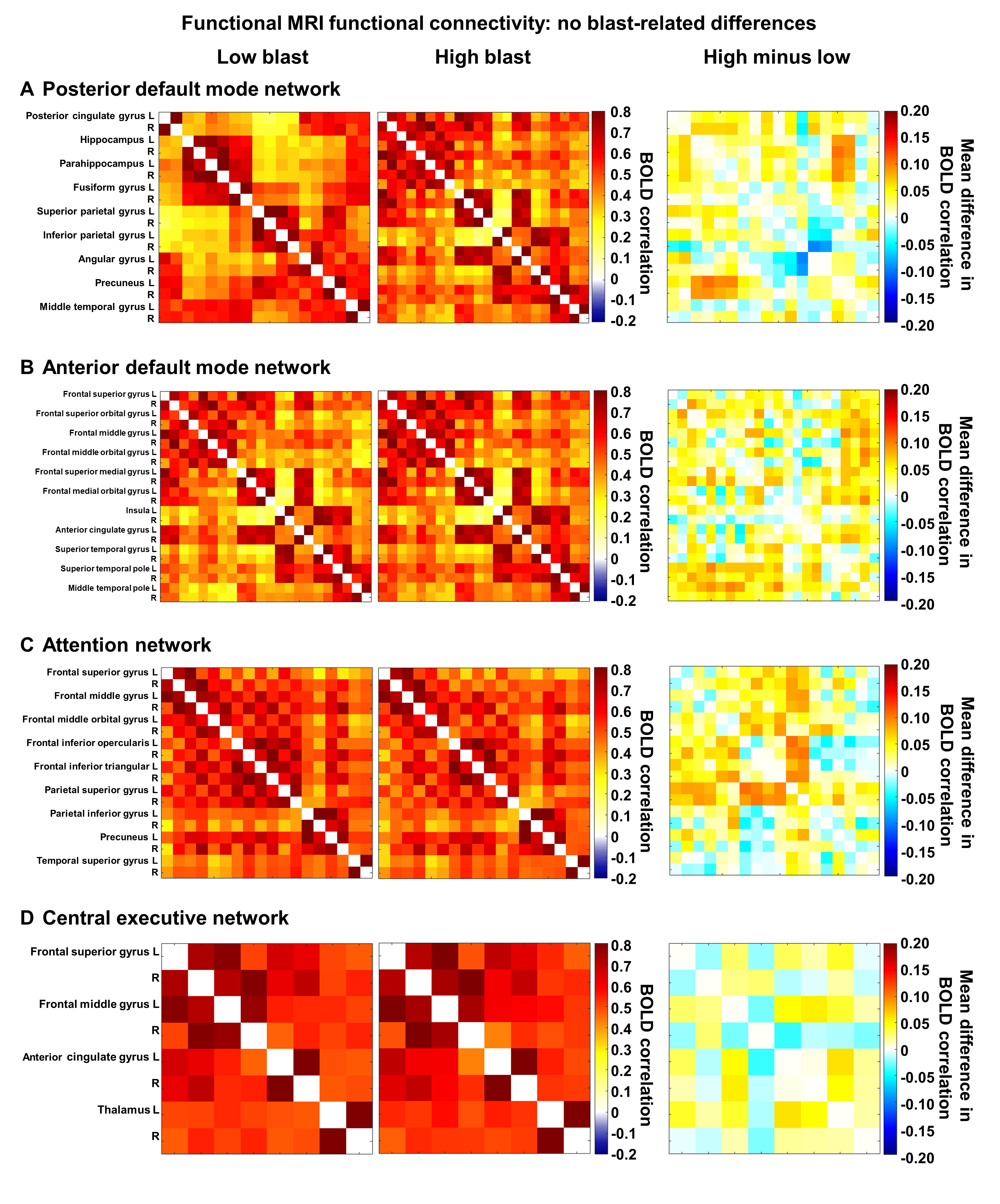


**Supplementary Figure 9:** Functional connectivity matrices scaled by BOLD correlations indicated by resting-state functional MRI in the (**A**) posterior (pDMN) and (**B**) anterior default mode networks (aDMN), (**C**) attention network (AN), and (**D**) central executive control network (CEN) with the mean connectivity for individuals with (**first column**) low blast exposure and (**second column**) high blast exposure, and **(third column**) the group difference. Network-based statistic analyses with F-tests revealed that there were no blast-related differences in (**A**) pDMN, (**B**) aDMN, (**C**) AN, or (**D**) CEN functional connectivity as measured by functional MRI. Warm elements in the high and low blast group mean matrices indicate higher connectivity and cool colours indicate lower connectivity; warm elements in the high minus low blast group difference matrices indicate higher connectivity in the high blast group and cool colours indicate lower connectivity in the high blast group relative to the low blast group.


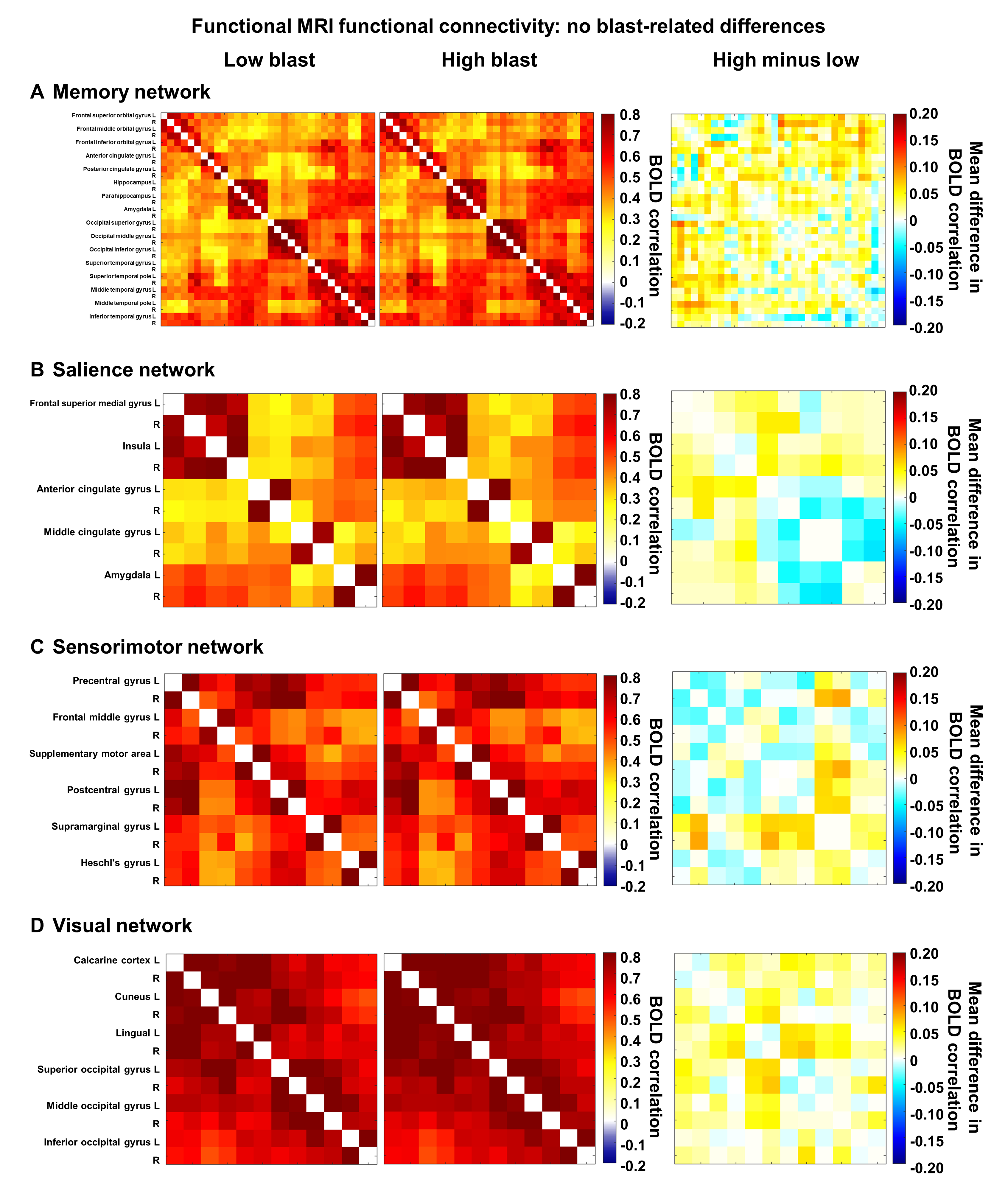


**Supplementary Figure 10:** Functional connectivity matrices scaled by BOLD correlations indicated by resting-state functional MRI in the (**A**) memory network (MN), (**B**) salience network (SN), (**C**) sensorimotor network (SMN), and (**D**) visual network (VN) with the mean connectivity for individuals with (**first** **column**) low blast exposure and (**second** **column**) high blast exposure, and (**third** **column**) the group difference. Network-based statistic analyses with F-tests revealed that there were no blast-related differences in (**A**) MN, (**B**) SN, (**C**) SMN, or (**D**) VN functional connectivity as measured by functional MRI. Warm elements in the high and low blast group mean matrices indicate higher connectivity and cool colours indicate lower connectivity; warm elements in the high minus low blast group difference matrices indicate higher connectivity in the high blast group and cool colours indicate lower connectivity in the high blast group relative to the low blast group.
